# Optical read-out of Coulomb staircases in a moiré superlattice via trapped interlayer trions

**DOI:** 10.1038/s41565-021-00970-9

**Published:** 2021-09-23

**Authors:** Hyeonjun Baek, Mauro Brotons-Gisbert, Aidan Campbell, Valerio Vitale, Johannes Lischner, Kenji Watanabe, Takashi Taniguchi, Brian D. Gerardot

**Affiliations:** 1 Institute of Photonics and Quantum Sciences, SUPA, Heriot-Watt University, Edinburgh, UK; 2 Departments of Materials and Physics and the Thomas Young Centre for Theory and Simulation of Materials, Imperial College London, London, UK; 3 Research Center for Functional Materials, National Institute for Materials Science, Tsukuba, Japan; 4 International Center for Materials Nanoarchitectonics, National Institute for Materials Science, Tsukuba, Japan

**Keywords:** Two-dimensional materials, Quantum optics, Quantum optics

## Abstract

Moiré patterns with a superlattice potential can be formed by vertically stacking two layered materials with a relative twist or lattice constant mismatch. In transition metal dichalcogenide-based systems, the moiré potential landscape can trap interlayer excitons (IXs) at specific atomic registries. Here, we report that spatially isolated trapped IXs in a molybdenum diselenide/tungsten diselenide heterobilayer device provide a sensitive optical probe of carrier filling in their immediate environment. By mapping the spatial positions of individual trapped IXs, we are able to spectrally track the emitters as the moiré lattice is filled with excess carriers. Upon initial doping of the heterobilayer, neutral trapped IXs form charged IXs (IX trions) uniformly with a binding energy of ~7 meV. Upon further doping, the empty superlattice sites sequentially fill, creating a Coulomb staircase: stepwise changes in the IX trion emission energy due to Coulomb interactions with carriers at nearest-neighbour moiré sites. This non-invasive, highly local technique can complement transport and non-local optical sensing techniques to characterize Coulomb interaction energies, visualize charge correlated states, or probe local disorder in a moiré superlattice.

## Abstract (web summary)

Moiré-trapped interlayer excitons in a transition metal dichalcogenide heterobilayer serve as a sensitive optical probe of carrier filling in their immediate environment to characterize the doping of the moiré superlattice.

## Main

Van der Waals heterostructures can be designed to confine electrons and holes in unique ways. One remarkable approach is to vertically stack two atomically thin layers of transition metal dichalcogenide (TMD) semiconductors. The relative twist or lattice mismatch between the two layers leads to moiré pattern formation, which modulates the electronic band structure according to the atomic registry. Single-particle wave packets can be trapped in the moiré-induced potential pockets with three-fold symmetry, leading to the formation of trapped interlayer excitons (IXs)^1,2^. Trapped IXs, observed so far in MoSe_2_/WSe_2_ heterobilayers, have compelling properties. In the limit of low temperature and weak excitation, the confocal photoluminescence (PL) spectra exhibit a few sharp lines (~100 μeV linewidths) with strong helical polarization dependent on the atomic registry and the ***C***_**3**_ symmetry of the crystal lattice^3,4^. In addition, highly uniform *g*-factors dependent on the relative layer twist are observed, clear fingerprints of the spin and valley configurations of excitons composed of band edge electrons and holes^3–6^. The PL emission from an individual trapped IX exhibits photon antibunching due to its quantum nature and, due to its large out-of-plane permanent dipole, is highly tunable in energy with a vertical electric field^7^. With increasing excitation power, the trapped IX density increases and the ensemble emission exhibits broader linewidths (≥5 meV) and multiple peaks due to different IX species can arise^8–12^, including charged excitons (trions), which can be controlled in gate-tunable devices^9,10,12^.

However, it is not yet clear what the relationship of the trapped IXs with the moiré superlattice is. In principle, trapped IXs should form in regular arrays according to the moiré pattern^1,2^. But, because the superlattice periodicity is ~10 nm, visualizing emitter positions in the far field is a substantial challenge. Nevertheless, far-field optical spectroscopy has revealed the existence of intralayer moiré excitons and hybridization^13,14^ and enabled observations of strong electron and hole correlations^15–18^, providing evidence that the moiré superlattice can be robust at the micrometre-scale in TMD heterobilayers. At the nanoscale, however, reconstructions, strain, ripples and other imperfections can affect moiré patterns, as observed with non-destructive local imaging techniques^19,20^. These structural ‘imperfections’ in a moiré pattern can have considerable impact on the optical properties of IXs^21–23^. But, for trapped IXs that exhibit magneto-optical properties consistent with perfect ***C***_**3**_ symmetry at a specific atomic registry, it is unclear how robust the moiré superlattice is and what the local environment of a trapped IX might be.

A trapped exciton can form a highly sensitive electrometer with optical read-out^24,25^. In this study we have exploited this technique to probe the local charge environment of trapped IXs as a function of carrier doping. We show that the trapped IX emission energy is sensitive to the Coulomb interaction energy of single nearby electrons trapped in the plane of the TMD.

### Electrostatic doping in a moiré superlattice

**Fig. 1:**
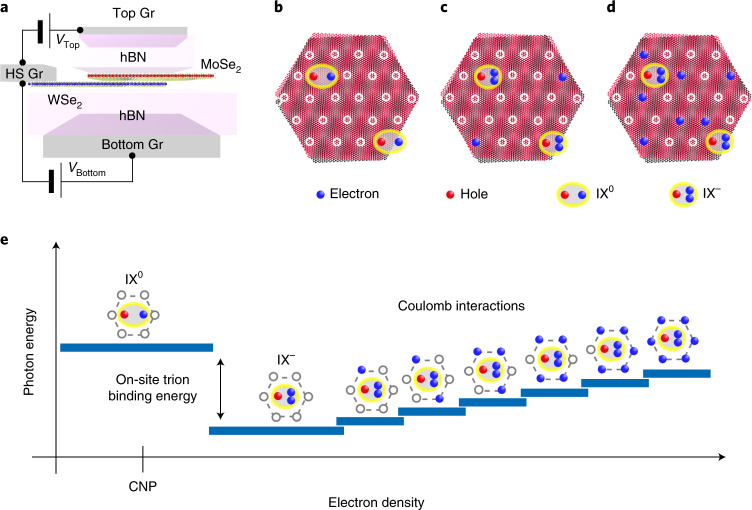
Schematic illustration of moiré lattice doping. **a**, A schematic of the dual-gated device structure for independent control of the electric field and heterobilayer doping. Gr, graphene; HS, heterostructure. **b**, A few IXs confined in moiré trapping sites in a 2H-type stacked MoSe_2_/WSe_2_ heterobilayer. The moiré trapping sites are shown as white circles. The key for the other elements contained in the superlattice are shown below **b**–**d**. **c**,**d**, With increasing doping of the moiré superlattice, IX trions are formed (**b**) and the number of filled moiré lattice sites increases (**d**). **e**, Illustration of the trapped IX energy versus electron density. As the electron density increases, a neutral IX converts into an on-site IX trion and the photon emission jumps to a lower energy. Successive filling of NN moiré sites leads to a Coulomb staircase.

Our concept is illustrated in Fig. 1. Figure 1a shows a sketch of the dual-gated 2H-type stacked MoSe_2_/WSe_2_ heterostructure device that allows independent control of doping and electric field. Figure 1b–d depicts a few moiré-trapped IXs at different electron doping densities in the moiré lattice. The white circles represent moiré sites where the hexagon centres of MoSe_2_ and WSe_2_ are vertically aligned (that is, with an $H_h^h$ local atomic registry). At low excitation power, only a few optically generated IXs are formed and confined in the most favourable moiré sites^12^, as shown in Fig. 1b. As additional electrons are added, the neutral IXs (IX^0^) are charged, forming on-site negative trions (IX^–^), and more empty moiré sites start to fill with excess electrons (Fig. 1c). Upon further electron doping, the number of moiré trapping sites filled by excess electrons increases and the cumulative Coulomb interaction between the trapped IXs and the spatially pinned electrons becomes stronger (Fig. 1d). Figure 1e shows a schematic of the expected change in photon energy for a trapped IX as increasing electron density leads to sequential filling of the six nearest-neighbour (NN) moiré sites. At small electron densities, the Fermi level in the device stays within the bandgap and the energy of the localized IX^0^ remains constant, resulting in an energy plateau around the charge neutrality point (CNP). As the electron density increases and the Fermi energy reaches the bottom of the conduction band, excess electrons start to fill the moiré lattice. The first electrons are expected to occupy the same moiré sites as the trapped IXs due to the positive on-site trion binding energy^9,10,12^. This leads to the formation of an on-site IX^–^, whose energy is redshifted relative to the trapped exciton. Upon further electron doping, the increasing density of excess electrons leads to a sequential filling of the NN sites. The repulsive Coulomb interaction between the IX^–^ and the electrons in the NN sites induces a blueshift of the IX^–^ energy. In contrast to conventional semiconductors, in which the doped carriers can move freely, the spatial pinning of the additional electrons to the moiré superlattice is expected to result in a discretization of the Coulomb interactions, creating an effective Coulomb blockade effect. Thus, the discrete Coulomb interactions between the localized IX^–^ and the carriers trapped in neighbouring moiré sites should give rise to a staircase-like blueshift of the exciton energy (Fig. 1e), rather than a continuous and smooth shift. We refer to this effect as the Coulomb staircase. These distinguishing spectral features under controlled carrier doping might represent valuable optical probes to read out the charge configuration of the local moiré landscape in TMD heterobilayers.

Here, we used the same dual-gated 2H-type stacked MoSe_2_/WSe_2_ heterostructure device used in previous works^7,12^. Hexagonal boron nitride (hBN) layers were used as dielectric spacers, with near identical thickness (~20 nm, see ref. ^7^ for detailed information), and graphene was used as the electrical contact for the top, bottom and heterobilayer gates. The carrier density in the device can thus be controlled by applying the same voltage to the top (*V*_Top_) and bottom (*V*_Bottom_) gates, minimizing the electric field across the heterobilayer. The 2H stacking configuration was confirmed by the Landé *g*-factor of the trapped IXs, a clear indicator of the relative valley alignment between the layers hosting the carriers^3–6,12^. The quality of the sample and formation of the moiré superlattice were undoubtedly demonstrated by voltage-dependent reflectance spectroscopy measurements: clear signatures of strongly correlated states in both the conduction and valence bands were observed for fractional filling factors of 1/3, 2/3, 1 and 4/3 (Supplementary Sections 1 and 2). In-depth analysis of this experiment is beyond the scope of the current manuscript, but our observations are consistent with recent reports of correlated insulating states in angle-aligned WSe_2_/WS_2_ heterobilayer samples^15–18^. Based on the applied voltage required to realize the specific fractional filling factors, the relative twist angle was determined to be 56.7 ± 0.2° across the sample (seven independent positions were measured). This confirms our estimate of the twist angle from the cleaved edges of MoSe_2_ and WSe_2_ (see ref. ^7^ and Supplementary Section 3), and our expectation that moiré domain reconstruction is unlikely in our sample^26,27^.

### Charged moiré interlayer excitons

**Fig. 2:**
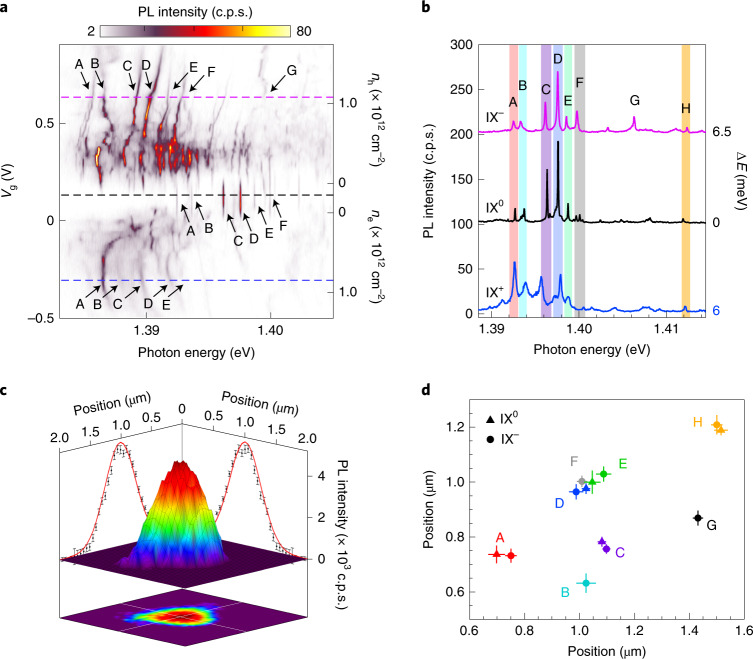
Doping dependence of moiré IXs. **a**, PL spectrum map as a function of *V*_g_. The prominent IX emission peaks are labelled as A–G in each doping region. The dashed lines identify the *V*_g_ of the linecuts for the spectra shown in **b**. *n*_e_, electron density; *n*_h_, hole density. **b**, Representative PL spectra for trapped IX^0^, IX^–^ and IX^+^. For direct comparison with the IX^0^ PL spectrum, the IX^–^ (IX^+^) PL spectra are shifted by 6.5 (6.0) meV in relative photon energy. **c**, A high-resolution spatial map of the integrated PL intensity of emitter D. From the Gaussian fits, the spatial position can be determined with ~25 nm precision. **d**, Spatial positions of the trapped IX^0^ and IX^–^ for various emitters. Error bars represent the standard deviation of repetitive measurements.

Figure 2a shows a confocal PL spectrum measured at a representative position in the sample (position P_1_) as a function of the applied gate voltage (*V*_g_ = *V*_Top_ = *V*_Bottom_). The energy of the continuous wave excitation laser was set to 1.705 eV to resonantly excite the 1*s* state of intralayer A excitons of WSe_2_, and an excitation power of 9 nW was used to ensure that only a handful of trapped IXs were optically generated^12^. At *V*_g_ = 0.1 V, the PL spectrum shows several discrete lines with emission energies in the range 1.39–1.40 eV, in agreement with previously reported values for localized IX^0^ in 2H-stacked MoSe_2_/WSe_2_ heterobilayers^3,4^. As expected for a device with symmetric gates, the emission energy of the trapped excitons remains constant in the neutral region (0.04 V < *V*_g_ < 0.19 V), confirming a negligible electric field in the direction parallel to the IX permanent electric dipole. We have labelled the peaks in this region A to F. At *V*_g_ > 0.19 V (that is, electron doping), the spectrum changes drastically: the overall emission energy redshifts abruptly by ~7 meV (in agreement with the formation of on-site IX^–^ trions^12^) and the number of emission peaks increases compared with the neutral charge regime (Supplementary Section 4). A further increase in *V*_g_ results in a combination of continuous and staircase-like blueshifts of the emission energies of the localized IX^–^. At *V*_g_ ≈ 0.4 V, the additional PL peaks collapse again into a handful of IX^–^ (labelled from A to G). At larger biases, the intensity decreases; we do not understand this behaviour but speculate that Auger processes could be involved. The behaviour in the hole-doping regime (negative applied *V*_g_), in which we observe the formation of positively charged IXs (IX^+^) with an average binding energy of ~6 meV, is generally similar to the n-doped regime. We remark that these spectra as a function of doping are remarkably reproducible: the spectral jumps and slopes observed as the doping is tuned are reproducible numerous times; they are unlikely to be caused by charge noise. Figure 2b shows the PL spectra at *V*_g_ = –0.3 (IX^+^), 0.1 (IX^0^) and 0.6 V (IX^–^) extracted from Fig. 2a (indicated by the dashed lines); each spectrum has been shifted in energy by the amount Δ*E* indicated on the right of the figure. Surprisingly, we observe that the number of emission peaks in the spectra of the charged IXs, their relative energies and their overall relative intensities resemble the PL spectrum observed in the neutral charge regime. We note that this behaviour is not particular to this position in the sample, but is a general behaviour observed at several other positions (Supplementary Section 5). Such similarities between the PL spectra under negative, neutral and positive doping conditions suggest that the PL peaks in the different doping regimes originate from the same moiré potential traps, whereas the spectral jumps as carriers fill the lattice are caused by each emitters’ local environment.

To corroborate this hypothesis, we employed a subdiffraction positioning technique to track the spatial positions of emitters A–H as a function of doping. In a confocal microscope, the spatial two-dimensional (2D) intensity distribution resulting from a subdiffraction point-like source (such as a localized IX) is given by the point spread function. The centre of the 2D intensity distribution corresponds to the spatial position of the emitter, which can be located with arbitrarily high precision under the appropriate experimental conditions^28,29^. Our method to determine the spatial positions of the trapped IXs involved several steps: first, we obtained 2 × 2 µm^2^ high-resolution spatial PL maps with a step size of 50 nm centred around the spatial position where the brightest emission is observed. Second, we obtained 2D spatial maps of the integrated PL intensity for each emitter. This was achieved by fitting the emission peak of each emitter to a Lorentzian function and plotting the resulting intensity as a function of the spatial position on the sample. We note that this process can be carried out efficiently as long as the different emitters can be spectrally resolved and present signal-to-noise ratios of >5. Figure 2c shows an example of the resulting spatial distribution of the integrated PL intensity from a single trapped IX, as obtained for emitter D under n-doped conditions. The position of each trapped exciton and its uncertainty (standard deviation) can then be obtained by numerical fitting of the corresponding spatial intensity distribution to a 2D Gaussian^30^. To mitigate the effects of possible experimental artifacts in the emitter positioning (such as scanning stage hysteresis or instabilities), the described localization procedure was repeated twice for each *V*_g_, and an average position and uncertainty were obtained for each trapped IX. This process allowed us to estimate the spatial positions of the individual trapped IXs with an average accuracy of 25 nm (that is, ~20 times smaller than the diameter of our confocal microscopy spot). Figure 2d shows the estimated spatial positions of the emitters shown in Fig. 2b under neutral and n-doping conditions. We note that the spectral overlap of peaks B and F at *V*_g_ = 0 V prevented us from extracting a reliable spatial position for these emitters in the neutral doping regime. The good agreement observed for both the individual and the relative positions of the emitters in the undoped and electron-doped regimes corroborates that the spectral lines observed in the neutral and n-doped conditions originate from the same moiré potential traps. Further, the spatial mapping of the trapped IX positions indicates that, at the exceptionally low density of excitons that we optically generated, their position is essentially random: the moiré lattice is not discernible. We speculate that the particular site chosen by the exciton is likely determined by the local environment and perhaps aided by dipolar repulsion effects^31,32^.

### Coulomb staircases in a moiré superlattice

**Fig. 3:**
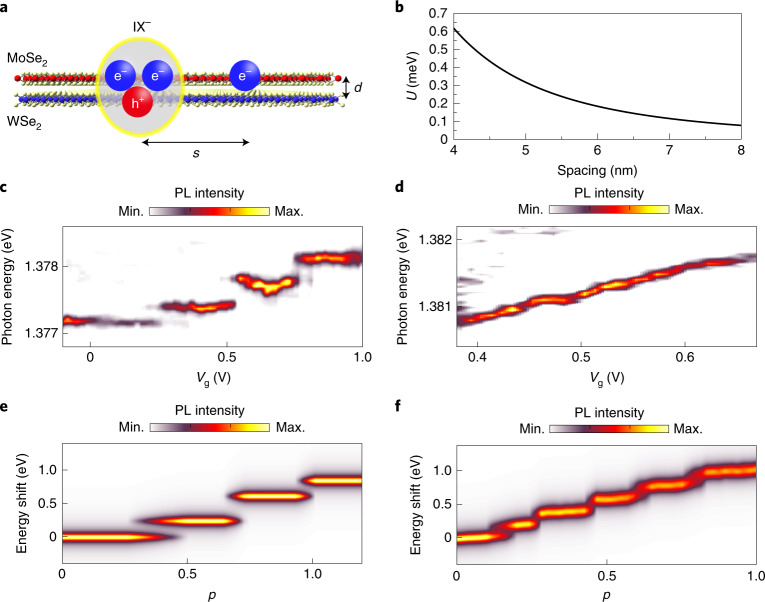
The Coulomb staircase. **a**, Side-view schematic of a trapped IX^–^ and one electron filled in a NN moiré site. Due to the interlayer distance between the hole and the electrons in the IX^–^, the Coulomb interaction between the IX^–^ and a NN electron is repulsive. **b**, The calculated Coulomb interaction energy between IX^–^ and a single NN moiré-trapped electron as a function of moiré lattice spacing. **c**,**d**, Two representative PL peak shift trends with electron doping. Discrete spectral jumps (**c**) or a continuous evolution with small jumps (**d**) are observed. **e**,**f**, Monte Carlo simulation results for discrete (**e**) and continuous (**f**) staircase changes. The simulation control parameter (*p*) was swept from 0 to 1 to simulate the increasing filling factor. With *p* = 0 (1), all moiré sites were set to be empty (filled). Min., minimum; Max., maximum.

Having determined that the PL emission peaks observed at *V*_g_ = 0.1 and 0.6 V in Fig. 2a belong to the same moiré-trapped IXs, we next focused on the behaviour of the emission energy of IX^–^ as a function of electron doping in several spatial positions of the sample. As previously discussed for Fig. 2a, the emission energy of the trapped IX^–^ blueshifts with increasing electron doping, featuring a combination of staircase-like and continuous evolution. We speculate that such distinct spectral features have their origin in the Coulomb interaction between the localized IX^–^ and electrons trapped in neighbouring moiré sites. To investigate this in more detail, we modelled the Coulomb interaction between a trapped IX^–^ and a nearby, spatially pinned, excess electron. Figure 3a shows a schematic illustration of the real-space configuration of the charges considered in our calculation. The Coulomb interaction (*U*) between the localized IX^–^ and the nearby electron modifies the energies of both the initial (IX^–^) and final states (single electron) of the PL emission process. It can therefore be estimated by the equation:

1 $$U = \frac{{e^2}}{{4\uppi \varepsilon _{\mathrm{r}}\varepsilon _0}}\left[ {\frac{1}{s} - \frac{1}{{\sqrt {s^2 + d^2} }}} \right],$$

where *e* is the elementary electron charge, *ε*_0_ is the dielectric permittivity of a vacuum, *ε*_r_ = 4.5 is the relative dielectric constant of hBN^33,34^, *s* is the moiré period and *d* = 0.5 nm is the interlayer distance. Moreover, because the thickness of the hBN spacers in our sample (~20 nm) is much larger than the estimated *s* we ignored the small screening effect caused by the conducting graphene gates. Figure 3b shows the calculated *U* as a function of *s*. Because *d* is much smaller than *s*, *U* decreases as ~1/*s*^2^.

Figure 3c,d presents two representative examples of the energy blueshift observed for IX^–^ in our heterobilayer as the excess electron density increases (see Supplementary Section 6 for several more examples). As can be seen in Fig. 3c and Supplementary Section 6, increasing *V*_g_ leads to a staircase-like blueshift of the emission energy for some IX^–^, featuring well-defined discrete spectral jumps. We note that these spectral features are highly reproducible upon electron doping (for example, multiple scans in *V*_g_). The observed energy jumps are in the range of 0.2‒0.4 meV, which corresponds to a moiré period of 4.6–5.8 nm and agrees very well with the 5.7 ± 0.4 nm moiré lattice estimated from the twist angle of our heterobilayer (Supplementary Section 2). These results support the hypothesis that the staircase-like blueshifts of IX^–^ originate from the Coulomb interactions of the localized excitons with charge carriers trapped in NN sites.

We underpinned the experimental observation of the Coulomb staircases by a Monte Carlo simulation of the IX trion energy as the moiré lattice sites are randomly occupied with electrons (see Methods section). The model successfully reproduced the prominent features of the experimental data, as exhibited in Fig. 3e,f, and could be used to gain further insights into the local environment of each trapped IX. In the following, we provide a brief summary of these findings. (1) The Coulomb interaction between the IX and NN sites dominates the trapped IX emission energy and accounts for the spectral jumps. (2) The preferred sequential filling of the NN sites results in reduced overlap of the different energy plateaux. (3) Inhomogeneities in the spectral jump energy is caused by small (nanometre scale) variations in the moiré period for an individual NN site. (4) The voltage extent of each charging plateau in the energy staircase is given by the probability of occupation of the corresponding NN site. (5) Because *U* decreases as ~1/*s*^2^, a long-range Coulomb interaction with a single electron occupying a non-NN site has a small effect. However, the sum of such interactions from filling many non-NN sites leads to a continuous evolution (as shown in Fig. 3d,f). We refer to Supplementary Section 7 for full details about the model.

### Polarization of charged moiré interlayer excitons

**Fig. 4:**
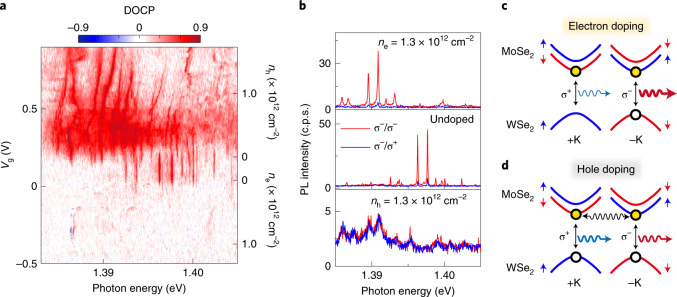
DOCP as a function of doping. **a**, DOCP map as a function of *V*_g_. The map was obtained at the same spatial position as the data in Fig. 2. **b**, Representative PL spectra for each doping condition. The red and blue lines represent co-polarized and cross-polarized PL emission, respectively, in each spectrum. A DOCP of nearly 0 is observed with hole doping. **c**,**d**, Energy band diagrams for IX in electron-doping (**c**) or hole-doping (**d**) conditions with σ^–^ excitation, respectively. For hole doping, intervalley scattering of the electron is represented by a wavy arrow.

To further investigate the properties of the individual trapped IXs, we monitored their degree of circular polarization (DOCP) as a function of carrier doping. We define the DOCP as $\frac{{I_{\upsigma ^ - } - I_{\upsigma ^ + }}}{{I_{\upsigma ^ - } + I_{\upsigma ^ + }}}$, where $I_{\upsigma ^ + }\,(I_{\upsigma ^ - })$ is the intensity of right (left) circularly polarized emission. Figure 4a shows the DOCP as a function of *V*_g_ obtained for position P_1_ under σ^–^ polarized laser excitation. Representative PL spectra with σ^+^ and σ^–^ collection polarization are presented in Fig. 4b for n-doped, undoped and p-doped conditions. In Fig. 4a, the doping map shows almost no charge noise and is highly reproducible. The exception is one trapped IX^+^ emitter, which appears as blue and red peaks at ~1.3866 eV in Fig. 4a due to a slightly different energy in the σ^+^ and σ^–^ maps. We observe that the trapped IXs exhibit strong co-polarization with the excitation laser for both the undoped and electron doping conditions, with an estimated DOCP of 0.7 (0.86) at *V*_g_ = 0.1 (0.56 V). In stark contrast, the DOCP of the trapped IXs reduces to almost 0 in the hole-doped regime. We interpret the drastic change in the DOCP for hole doping as an indication that the photoexcited holes in WSe_2_ preserve the valley polarization of the excitation laser, whereas the valley polarization of the excitation laser field is lost in the photoexcited electrons. Figure 4c,d shows schematic configurations of IX^–^ and IX^+^ under σ^–^ excitation for a 2H-type stacked heterobilayer. Due to the type-II band alignment, the excess electron (hole) of IX^–^ (IX^+^) resides in the MoSe_2_ (WSe_2_) layer. As a consequence of Pauli exclusion, the additional carrier occupies the opposite valley to that containing the initial IX^0^. Therefore, because electrically injected electrons (holes) populate both +K and –K valleys for IX^–^ (IX^+^), the DOCP of the trion state is given by the conservation of the valley polarization of the optically created hole (electron) in WSe_2_ (MoSe_2_). The large DOCP values observed for IX^–^ in the electron-doped regime suggest that the valley polarization of the excitation laser can be robustly preserved by the excited hole in WSe_2_. In contrast, the absence of DOCP for hole-doping indicates that the valley polarization of the excitation laser is totally lost for the photoexcited electrons in MoSe_2_. Such contrasting behaviour for the photoexcited carriers in MoSe_2_ and WSe_2_ originates from the different intervalley scattering processes in these materials. As previously reported, the valley relaxation time in monolayer MoSe_2_ is much shorter than that in WSe_2_, leading to valley depolarization^35,36^. Additionally, the slight increase in DOCP from 0.79 for IX^0^ to 0.86 for IX^–^ can also be explained by the higher scattering rate in MoSe_2_. For example, in the case of σ^–^ excitation, the probability of occupancy of the conduction band at the –K valley for IX^0^ decreases due to the intervalley scattering of electrons in MoSe_2_ limiting the recombination rate of IX at the –K valley. On the other hand, for IX^–^, the scattered electrons can be compensated by electrically doped electrons, and the recovered recombination rate leads to an increased DOCP because it is proportional to 1/(1 + *τ*/*τ*_r_), where *τ* is the exciton decay time and *τ*_r_ is the valley relaxation time^37^. Moreover, it is worth noting that the overall behaviour of the DOCP is similar under resonant excitation of MoSe_2_ A excitons (Supplementary Sections 9 and 10). This suggests that the valley polarization is preserved exclusively by the holes in WSe_2_ even under resonant excitation to MoSe_2_.

### Magneto-optical properties of charged moiré interlayer excitons

**Fig. 5:**
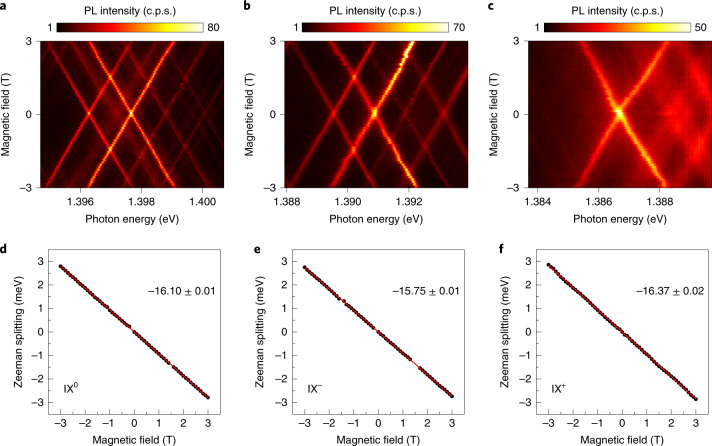
Magneto-PL characteristics of neutral and charged IXs. **a**–**c**, The PL spectra of IX^0^ (**a**), IX^–^ (**b**) and IX^+^ (**c**) (*V*_g_ = 0, 0.6 and –0.4 V, respectively) as the magnetic field is swept from –3 to 3 T. The excitation laser and PL collection were both linearly polarized. **d**–**f**, Plots of the Zeeman splitting versus magnetic field for IX^0^ (**d**), IX^–^ (**e**) and IX^+^ (**f**), yielding *g*-factors of –16.10, –15.75 and –16.37, respectively.

Finally, we investigated the magneto-optical properties of the localized IX under different doping conditions. Figure 5a–c shows the PL spectra of the trapped IXs at position P_1_ for applied *V*_g_ of 0 (IX^0^), 0.6 (IX^–^) and –0.4 V (IX^+^), respectively, as a function of the applied out-of-plane magnetic field (Faraday geometry). We observe that, similar to the behaviour reported for localized IX^0^ (refs. ^3,4,7^), both IX^+^ and IX^–^ show linear Zeeman splittings with no observable fine structure. Linear fits of the measured Zeeman splittings revealed Landé *g*-factors of –16.10, –15.75 and –16.37 for IX^0^, IX^–^ and IX^+^, respectively, as shown in Fig. 5d–f, which agree well with the effective *g*-factor expected for spin–triplet optical transitions in 2H-stacked MoSe_2_/WSe_2_ heterobilayers^4,6^.

## Conclusions

In summary, we have reported here the deterministic creation of a low density of moiré-trapped IX trions in a charge-tunable 2H-type stacked MoSe_2_/WSe_2_ heterostructure device. We spectroscopically investigated their properties and exploited their sensitivity to their immediate environment to characterize the doping of the moiré superlattice. First, by spatially tracking their positions, we were able to spectrally track individual emitters as a function of doping. This enabled us to unambiguously identify the on-site trion binding energy, which is highly uniform for numerous emitters, and probe the spectral jumps that arise with filling of the moiré superlattice. We observed several examples of the Coulomb staircase: stepwise changes in the IX trion emission energy due to Coulomb interactions with carriers at NN moiré sites. We performed a Monte Carlo simulation to better understand the effects of short-range moiré site uniformity and long-range interactions. These results demonstrate a non-invasive, highly local technique to characterize the moiré superlattice. An interesting prospect would be to combine this approach with complementary techniques to probe the local disorder, homogeneity and reconstruction in a moiré lattice. For example, the two types of trapped IX trion peak shifts in Fig. 3c,d show either well-defined discrete spectral jumps or a more continuous energy shift upon doping, respectively. These two general responses suggest that the local lattice in the vicinity of the trapped IXs has different properties, likely caused by disorder. For the IX peak in Fig. 3c, the small effect of long-range Coulomb interactions from a single electron occupying non-NN sites is negligible, which implies that the moiré site responsible for the IX trapping and its nearby sites have a much higher probability of charge occupation than sites farther away. On the other hand, moiré sites in the region of the emitter in Fig. 3d can be considered quite homogeneous, allowing random charge filling of both NN sites and non-NN sites with equal probability. Combining this optical read-out technique with other surface probe microscopy techniques that provide structural information to correlate distinct features of local disorder (for example, strain, ripples, reconstruction and other imperfections) in a moiré superlattice^21,23,38^ would provide valuable new insights into moiré systems. Furthermore, an exciting prospect would be to locally probe long-range charge-ordered states in a fractionally filled moiré lattice, such as correlated Mott insulators and Wigner crystals^15–17^. The optical read-out of Coulomb interactions between a single moiré-trapped IX and its neighbouring charges presents a viable route to visualize the formation and Coulomb interaction energies of such charge-ordered states in TMD heterobilayers, although the signatures can be complex (Supplementary Section 8).

## Methods

### PL spectroscopy

A confocal microscope with an objective lens with a numerical aperture of 0.82 was used for PL measurements. The device was loaded into a closed-cycle cryostat (4 K) with a superconducting magnet. A continuous wave Ti:sapphire laser was used to resonantly excite MoSe_2_ and WSe_2_ A excitons at *λ* = 759.6 and 727.0 nm, respectively. The PL emission was dispersed in a 500-mm focal length spectrometer and detected by a nitrogen-cooled charge-coupled device with a spectral resolution of ∼70 μeV at *λ* = 900 nm for 1,200 lines mm^–1^. Polarization-resolved PL was measured by changing the relative angle between quarter-wave plate and linear polarizer both for laser excitation and PL collection.

### Monte Carlo simulation for Coulomb staircases

We simulated the change in emission energy of a stationary trapped exciton as a function of carrier density using a Monte Carlo model. Our model shared many features with that of Houel et al.^25^. We note that we did not simulate the Hubbard model here, for example, we accounted for only *U*, not for the hopping probability *t*, and that our model did not take into account interactions between the excess electrons. The occupation of an electron at each trapping site *i* was determined randomly and the resulting *U*_*i*_ caused by the occupied lattice sites was calculated. A control parameter (*p*), swept from 0 to 1, was introduced to simulate the doping density. This value was multiplied with a weighting factor *w*_*i*_, which varied from moiré site to site and accounted for the probability of the different sites to trap the injected excess carriers due to the inhomogeneity among moiré sites. Then *w*_*i*_*p* was compared with a random number (*r*) in the range 0 ≤ *r* ≤ 1. If *w*_*i*_*p* ≥ *r*, the moiré site was considered to be filled by an electron, otherwise the site was considered to be empty. Therefore, all sites were filled when *p* reached 1. This process was conducted at each trapping site, and *r* was newly regenerated for every comparison. In this way, the occupation state of the neighbouring moiré trapping sites was established, and the total Coulomb energy (*U*_tot_) from the occupied sites was calculated as $U_{{\mathrm{tot}}} = \mathop {\sum }\limits_i U_i$, where

$$U_i = \frac{{e^2}}{{4\uppi \varepsilon _{\mathrm{r}}\varepsilon _0}}\left[ {\frac{1}{s_i} - \frac{1}{{\sqrt {s_i^2 + d^2} }}} \right],$$

as discussed in Eq. (1). A PL spectrum was then created assuming a Lorentzian line shape with a width of 100 µeV and a central energy *U*_tot_. This process was repeated *N* (typically 100–1,000) times at a given *p* resulting in *N* PL spectra, which were summed together to obtain a final PL spectrum. Finally, by changing *p* from 0 to 1 we were able to simulate PL spectra at different doping levels.

## Online content

Any methods, additional references, Nature Research reporting summaries, source data, extended data, supplementary information, acknowledgements, peer review information; details of author contributions and competing interests; and statements of data and code availability are available at 10.1038/s41565-021-00970-9.

## Supplementary information

Supplementary Sections 1–11, Figs. 1–21 and references.

## Data Availability

The data described in this paper and presented in the Supplementary Information are available online at https://researchportal.hw.ac.uk/en/persons/brian-d-gerardot/datasets/.
