# Supplementary material for: Optical read-out of Coulomb staircases in a moiré superlattice via trapped interlayer trions
Source: Nat Nanotechnol. 2021 Sep 23;16(11):1237–43. doi: 10.1038/s41565-021-00970-9 (PMC8592839; doi:10.1038/s41565-021-00970-9)
Supplement: Supplementary file 1 — Supplementary Sections 1–11, Figs. 1–21 and references. [file 41565_2021_970_MOESM1_ESM.pdf]

nature portfolio

https://doi.org/10.1038/s41565-021-00970-9

Supplementary information

Optical read-out of Coulomb staircases in a moiré superlattice via trapped interlayer trions

In the format provided by the authors and unedited

Supplementary Information for

Optical read-out of Coulomb staircases in a moiré superlattice via trapped interlayer trions

Hyeonjun Baek,[1,] Mauro Brotons-Gisbert,[1] Aidan Campbell,[1] Valerio Vitale,[2] Johannes Lischner,[2] K. Watanabe,[3] T. Taniguchi,[4] and B. D. Gerardot[1,]**

[1]Institute of Photonics and Quantum Sciences, SUPA, Heriot-Watt University, Edinburgh EH14 4AS, UK

[2]Departments of Materials and Physics and the Thomas Young Centre for Theory and Simulation of Materials, Imperial College London, South Kensington Campus, London SW7 2AZ, United Kingdom

[3]Center for Functional Materials, National Institute for Materials Science, 1-1 Namiki, Tsukuba 305-0044, Japan

[4]International Center for Materials Nanoarchitectonics, National Institute for Materials Science, 1-1 Namiki, Tsukuba 305-0044, Japan

*Corresponding author: HJBaek@kist.re.kr, B.D.Gerardot@hw.ac.uk

Contents

Section S1. Doping dependence of *intralayer* excitons in monolayer regions of the device

Section S2. Observation of strongly correlated electron and hole states in our device

Section S3. Determination of twist angle from optical images

Section S4. PL spectra at 0.3 < V_g < 0.4 for electron doping

Section S5. IX PL spectra at different doping conditions

Section S6. Discrete spectral jumps with doping

Section S7. Monte Carlo simulation for discrete spectral jumps (short-range charge ordering)

Section S8. Monte Carlo simulation of long-range charge-ordered states

Section S9. Excitation energy dependence of moiré IX emission

Section S10. DOCP map with resonant excitation to the intralayer A-exciton of $MoSe_2$ (1.632 eV)

Section S11. Energy band diagrams for IX for an applied B-field

Section S1. Doping dependence of *intralayer* excitons in monolayer regions of the device

The intralayer emission from hBN encapsulated monolayer $MoSe_2$ is monitored from a sample region where the $MoSe_2$ does not overlap with WSe_2 layer. Figure S1(a) shows this intralayer PL emission as a function of the applied voltage V_g. The neutral exciton peak (X^0) at ~1.645 eV is dominant at 0 V. As the device is doped with electrons (holes), the negatively (positively) charged trions X^- (X^+) appear at 1.617 eV (1.619 eV).

We also perform reflectance contrast measurements in monolayer regions of the device. We define the reflectance contrast as $\Delta R/R_0$, where $\Delta R = R_s - R_0$ (with R_s the reflected intensity from the sample and R_0 the reflected intensity from a region of the heterostructure with no TMD layers present). Figure S1(b) shows V_g-dependent measurements of $\Delta R/R_0$ from hBN encapsulated monolayer regions of the device, in which we have labelled the main excitonic features. For monolayer $MoSe_2$ at charge neutrality (V_g = 0 V), only the neutral intralayer A-exciton is present, which we label X_{Mo}^0. When electrons are injected into the monolayer (V_g > 0 V) the attractive polaron, X_{Mo}^{AP-}, is formed at lower energy than X_{Mo}^0. X_{Mo}^0 also blue-shifts and loses oscillator strength in agreement with the description of a repulsive polaron X_{Mo}^{RP-}. When the monolayer $MoSe_2$ is hole doped (V_g < 0 V), X_{Mo}^{RP+} and X_{Mo}^{AP+} are formed. Similarly, when the WSe_2 is hole-doped, X_W^{RP+} and X_W^{AP+} are also formed. In the electron doping regime for monolayer WSe_2, the repulsive polaron X_W^{RP+} is formed and attractive polaron formation is allowed in both spin-singlet $X_W^{AP-,S}$ and spin-triplet ($X_W^{AP-,T}$) configurations. The observed features in both the photoluminescence and broadband reflectance agree very well with theoretical and experimental literature reports and confirm the device is of high quality and works as expected[1-5].

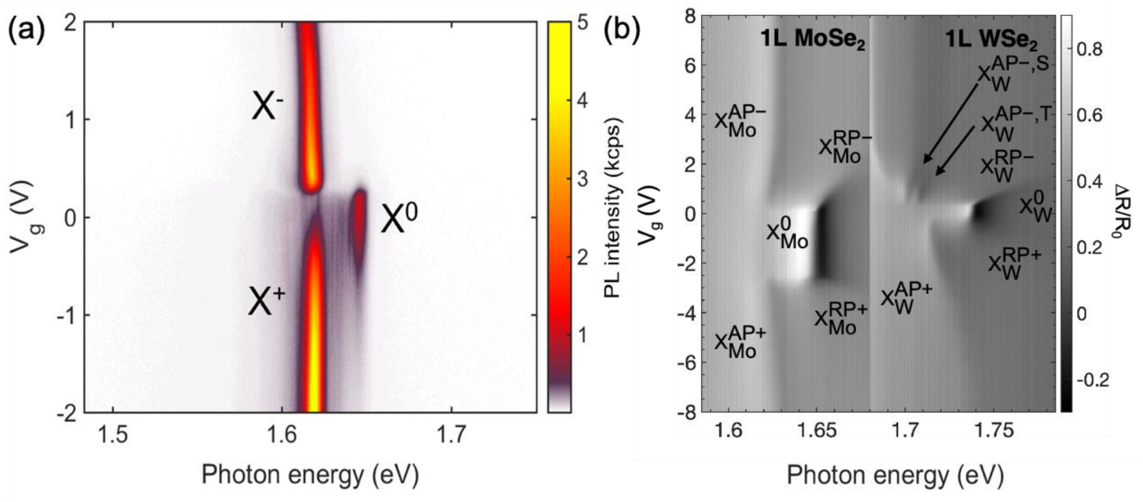

Figure S1. **(a)** PL of $MoSe_2$ intralayer excitons as a function of V_g. **(b)** Reflection contrast $\Delta R/R_0$ as a function of V_g in hBN encapsulated monolayer $MoSe_2$ and WSe_2 regions of the device.

Section S2. Observation of strongly correlated electron and hole states in our sample

The interpretation of our experimental results critically rests on the assumptions that the moiré superlattice exists in our sample and the device functions properly. In this section, we therefore perform differential reflection spectroscopy to provide additional experimental results that confirm the following:

1) The existence of the moiré superlattice in our sample, via the observation of strongly correlated states in both the conduction and valence bands.

2) The twist angle of our sample is 56.7 +/- 0.2 degrees, measured at 7 positions in the sample via application of the capacitor plate model to extract the moiré density at a filling factor of $\nu = -1$. This confirms the estimate from optical microscope images (see section S3).

We believe that these results provide confidence in the assumptions and interpretations we make in the main manuscript. However, an in-depth presentation of these results is beyond the scope of the current manuscript, so a small summary is provided in this section of the Supplementary Information.

We perform a doping sweep of the differential contrast $\Delta R/R_0$ in the 1L $MoSe_2$/1L WSe_2 (heterobilayer) region of the sample, as shown in Figure S2. At charge neutrality (V_g = 0 V), we clearly see the $MoSe_2$ and WSe_2 intralayer excitons ($X^0_{Mo,1}$ and X^0_{W}). When the sample is hole doped (V_g < 0 V), X^0_{W} loses oscillator strength. When electrons are injected into the sample (V_g > 0 V), $X^0_{Mo,1}$ quickly loses oscillator strength. These observations are consistent with Type-II band alignment[6-8]. In contrast to the monolayer $MoSe_2$ only region of the device (see section S1), a peak at higher energy than $X^0_{Mo,1}$ gains oscillator strength which we label $X^0_{Mo,2}$. We suggest the double excitonic feature arises from the formation of conduction moiré mini-bands which has been theoretically predicted for angle aligned WSe_2/$MoSe_2$ moiré heterostructures[9,10].

In both the electron and hole doping regimes, all species of intralayer excitons exhibit modulations in intensity, peak position, and linewidth not present in the monolayer regions. This agrees with experimental observations of the formation of moiré conduction and valence minibands[11,12]. Following the method demonstrated by Tang *et al.*[11], we identify –1.75 V as 1 hole per moiré lattice site using the integrated intensity peak of the X_W^0 in the hole doping regime (side panel of Fig. S2). Using the parallel plate capacitance model, the carrier concentration in the heterostructure can then be calculated using $n = \frac{\varepsilon\varepsilon_0\Delta V_g}{d_1} + \frac{\varepsilon\varepsilon_0\Delta V_g}{d_2}$ where ε is the permittivity of hBN, ΔV_g is the voltage offset between both the top and bottom gate and the heterostructure, and d_1 (d_2) is the thickness of the top (bottom) hBN layer (measured to be 17.4 ± 0.2 nm and 18.2 ± 0.3 nm respectively using nulling ellipsometry). For a small twist angle between two stacked layers, the moiré periodicity can be estimated using $\lambda_M = \frac{a_{Se}}{\sqrt{\delta^2+\theta^2}}$, where a_{Se} is the lattice constant of WSe_2, δ is the fractional lattice mismatch between the two layers and θ is the twist angle in radians.

For a triangular moiré pattern, the number of carriers required for one hole per site is given by $n_0 = 2\sqrt{3}\lambda_M^2$. Using the lattice constants of 3.280 Å and 3.288 Å for WSe_2 and $MoSe_2$, respectively[13], the twist angle is calculated to be 3.1 degrees, which agrees well with the angle estimated from the optical images (see section S3). Across the sample, we measure 7 spatial positions, and obtain 3.3 +/- 0.2 degrees, which corresponds to an average moiré period of 5.7+/- 0.4 nm.

Following the work by Jin *et al.*[14], we can use an empirical theoretical model to approximate the effect of this twist angle on the exitonic spectrum. Due to the moiré superlattice, the wavefunction from normally forbidden excitonic features mix with the bright exciton, gaining part of its oscillator strength. The splitting between the two excitonic features with highest oscillator strength can be estimated using $4E_M$, where E_M is the exciton kinetic energy in the first mini Brillouin zone, and is approximated by $E_M = \frac{\hbar b_i^2}{M}$, where b_j is the moiré reciprocal lattice vector and M is the exciton mass. The relative oscillator strength of the higher energy peak can then be estimated using $\left|\frac{V_j}{4E_M}\right|^2$, where V_j is the magnitude of the

moiré potential. From our measured twist angle, we use the model to estimate the splitting between the $MoSe_2$ excitons to be 40 meV.

Figure S3(a) shows the $\Delta R/R_0$ measured at charge neutrality (V_g = 0 V). For the fitting we model each excitonic feature using the real part of the Lorentzian oscillator function $f(E) = \frac{A(E_p^2 - E^2)}{(E_p^2 - E^2)^2 + E^2\gamma^2}$, where A is the amplitude, E_p is the central energy of the resonance and γ is the linewidth. From the fit we estimate the splitting between $X^0_{Mo,1}$ and $X^0_{Mo,2}$ to be 36 meV which is consistent with the theoretical prediction of 40 meV. Figure S3(b) shows a calculated $\Delta R/R_0$ spectrum using the predicted splitting, and a relative oscillator strength ratio of 5:1 between $X^0_{Mo,1}$ and $X^0_{Mo,2}$ which is set for good agreement between theory and experimental observations.

In order to better resolve the fractional filling modulations in intensity, position and linewidth of the excitonic features, we plot the doping dependence of the derivative of the reflection contrast with respect to applied gate voltage $d(\Delta R/R_0)/dV$, as shown in Figure S4. We observe abrupt changes in $d(\Delta R/R_0)/dV$ at every 1/3 fractional filling of the moiré superlattice for both electron and hole filling. This suggests that ordered correlated electron and hole states are formed at these values, similar to observations in WS_2/WSe_2 moiré heterostructures.[12,15]

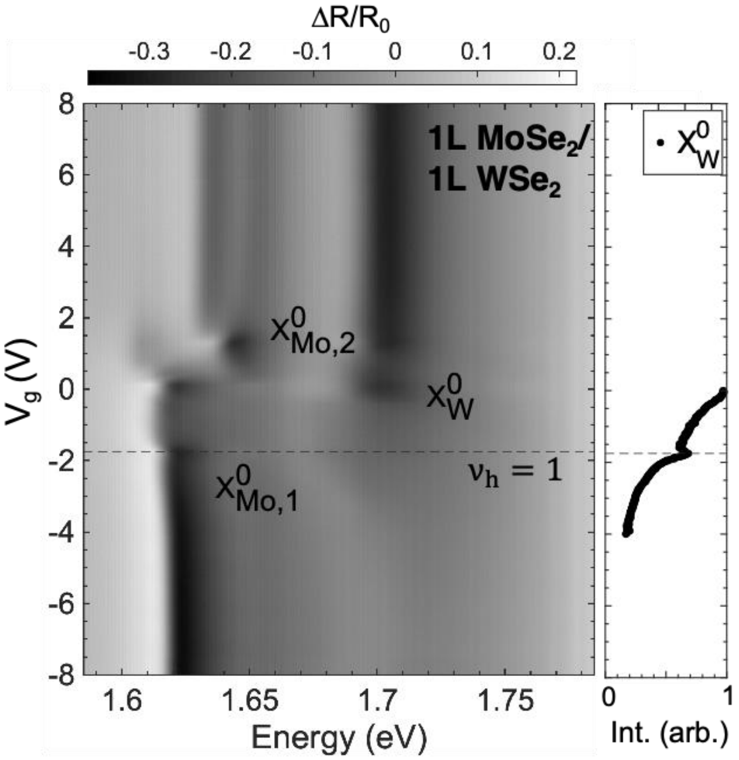

Figure S2. Doping sweep of $\Delta R/R_0$ in a representative 1L $MoSe_2$ /1L WSe_2 heterostructure region of the device. The side panel shows the integrated intensity of X_W^0 in the range 1.67-1.72 eV while the dashed line at –1.75 V indicates 1 hole per moiré lattice site ($\nu_h = 1$).

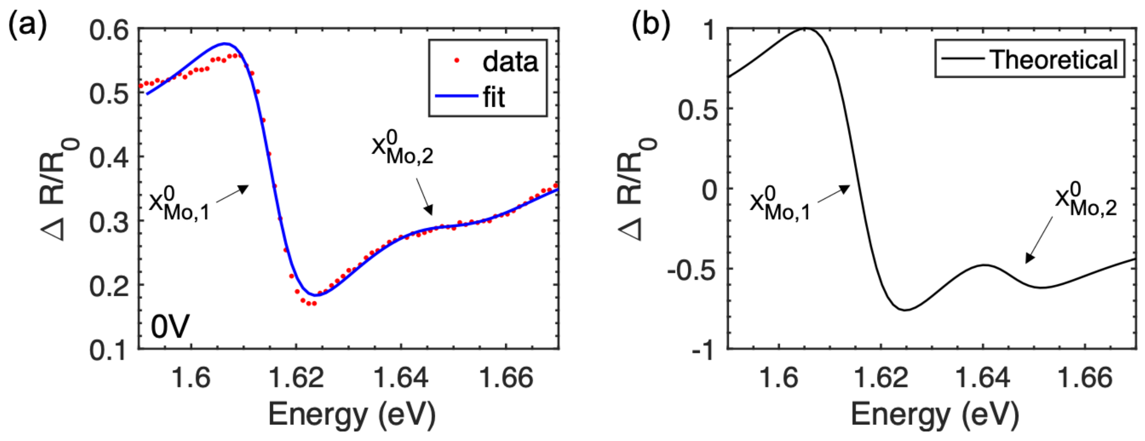

Figure S3. **(a)** Linecut of the differential contrast $\Delta R/R_0$ in the heterobilayer at charge neutrality. The solid line shows the fit to the experimental data, from which we extract a splitting of 36 meV between $X^0_{Mo,1}$ and $X^0_{Mo,2}$. **(b)** Theoretical curve of the differential contrast, based on the theoretically calculated splitting of 40 meV between $X^0_{Mo,1}$ and $X^0_{Mo,2}$. The relative oscillator strength of the two excitonic resonances was set to 5:1 for good agreement with the experimental data.

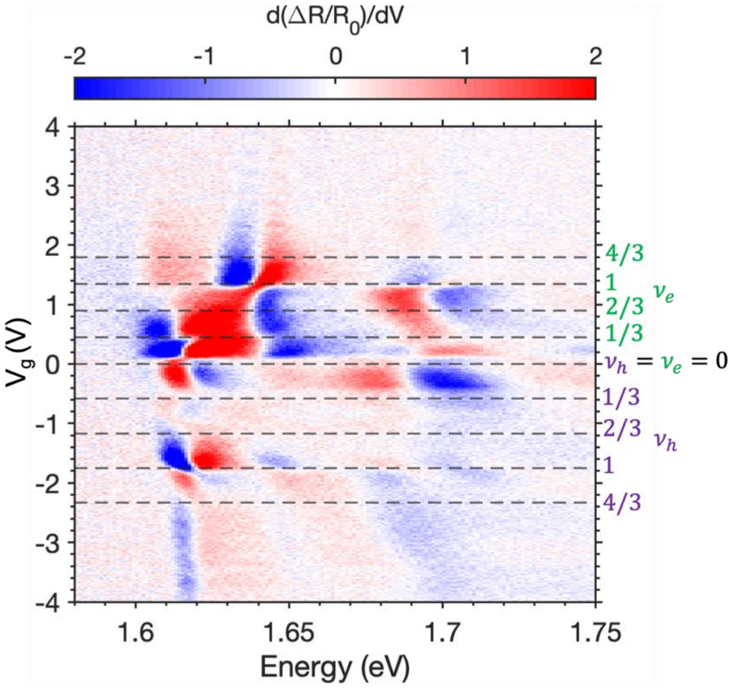

Figure S4. Doping dependence of the derivative of the reflection contrast with respect to the applied voltage (d(ΔR/R$_0$)/dV). The dashed lines indicate fractional fillings of the moiré superlattice with electrons (ν_e) or holes (ν_h). There are abrupt changes in d(ΔR/R$_0$)/dV at all labelled fractional fillings, indicating the presence of correlated electron and hole states.

Section S3. Determination of twist angle from optical images

Figure S5 shows an optical microscope image of the $MoSe_2/WSe_2$ heterostructure on PDMS. Cleaved edges of $MoSe_2$ and WSe_2 flakes are indicated by red and blue lines, respectively. From the relative angle between these edges, the twist angle for the heterostructure is estimated as 56.4°.

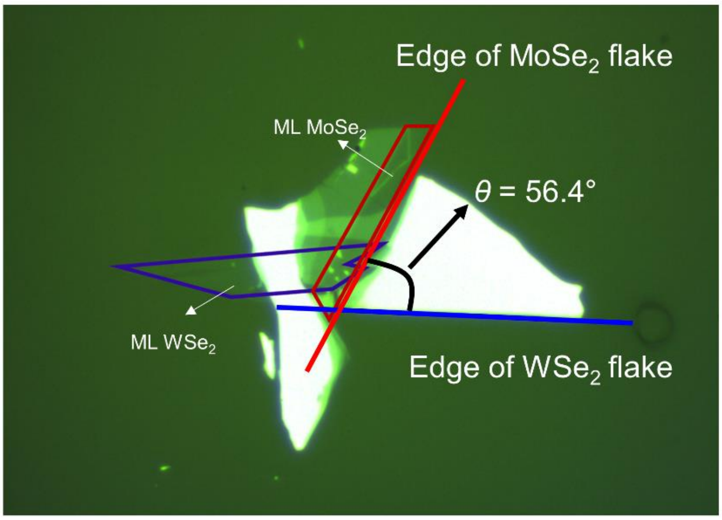

Figure S5. Optical microscope image of the $MoSe_2/WSe_2$ heterostructure on PDMS.

Section S4. PL spectra at 0.3 < V_g < 0.4 for electron doping

As shown in Fig. S6(a), the total integrated PL intensity of trapped IXs are several times brighter in the V_g range of 0.3–0.4 V than for V_g = 0 V or V_g > 0.4 V. This behaviour is not particular of the trapped IXs but can also be observed in the PL of the delocalised intralayer excitons of the individual TMD monolayers as shown in Fig. S6(b). The brighter PL emission of the excitons for V_g ~ 0.3–0.4 V allows us to observe additional PL peaks in this voltage range which otherwise are buried in the background of the PL spectra measured at V_g = 0 V and V_g > 0.4 V. This hypothesis is supported by the fact that the PL intensity of the 'additional' PL peaks decreases under further electron doping until it eventually vanishes into the PL spectrum background. Meanwhile, the PL emission energy of each trapped IX trion under electron doping depends on the particular charge configuration and filling order of its neighbouring sites (i.e., the local potential landscape of each emitter), which gives rise to different spectral behaviours. We believe that the combination of additional PL peaks and the different spectral behaviour of each PL peak result in more complex PL spectrum in the V_g range ~ 0.3–0.4 V.

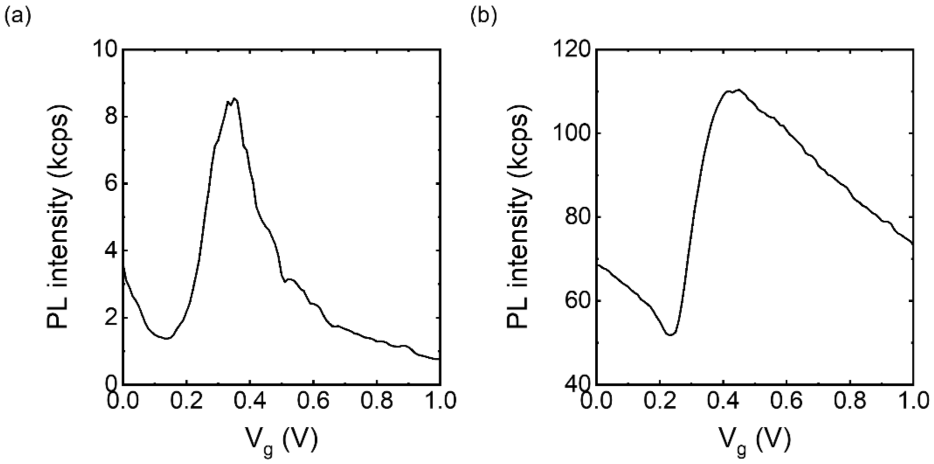

Figure S6. Plots of the integrated PL intensity versus V_g for moiré IXs shown in Fig. 2a in the main text **(a)** and for delocalised intralayer excitons in $MoSe_2$ shown in Fig. S1 **(b)**.

Section S5. IX PL spectra at different doping conditions

Figure S7 shows further representative IX PL spectra measured at different spatial positions with *n*-doped, undoped, and *p*-doped conditions. As stated and shown in the main text, the PL profiles at each doping condition are very similar. The relative energies and intensities between peaks in each PL spectra are preserved at different doping conditions. These results provide further evidence that the red-shifted peaks in doped regions originate from the same emitters in the neutral region.

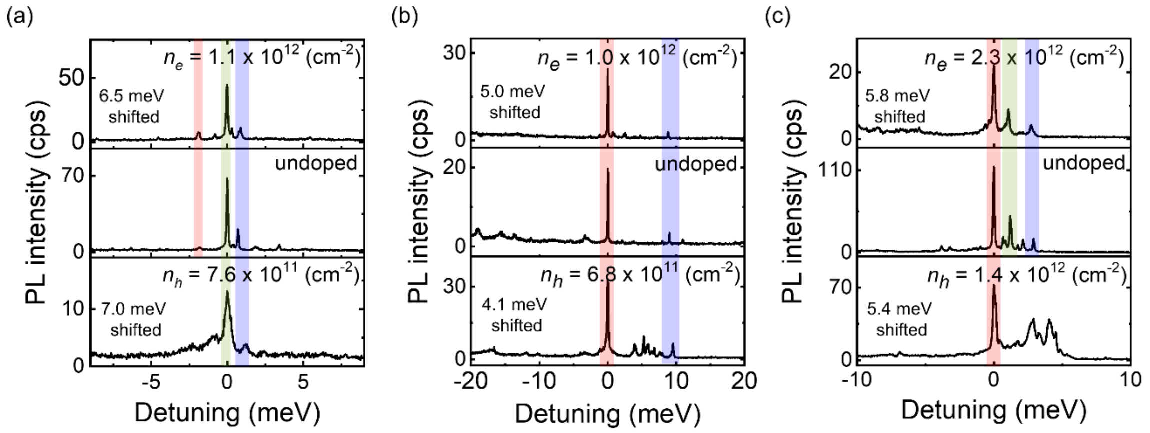

Figure S7. (a)-(c) PL spectra of moiré-trapped IXs measured at different spatial positions of the heterobilayer with *n*-doped (top panels), undoped (middle panels), and *p*-doped (bottom panels) conditions. PL spectra with *n*- and *p*-doped conditions are shifted 6.5 and 7.0 meV in (a), 5.0 and 4.1 meV in (b), and 5.8 and 5.4 meV in (c), respectively, to align them with the corresponding PL spectra with charge neutral conditions.

Section S6. Discrete spectral jumps with doping

Figures S8-S12 show PL maps as a function of electron density measured at different positions along the device. Zoomed images for regions indicated by rectangular boxes are added to display the trends of the peak shifts more clearly. The data shown in Fig. S8 is measured from position P_1, where the data for main text was obtained. Here, most of the peaks show a blue-shift with discrete steps as the electron density increases.

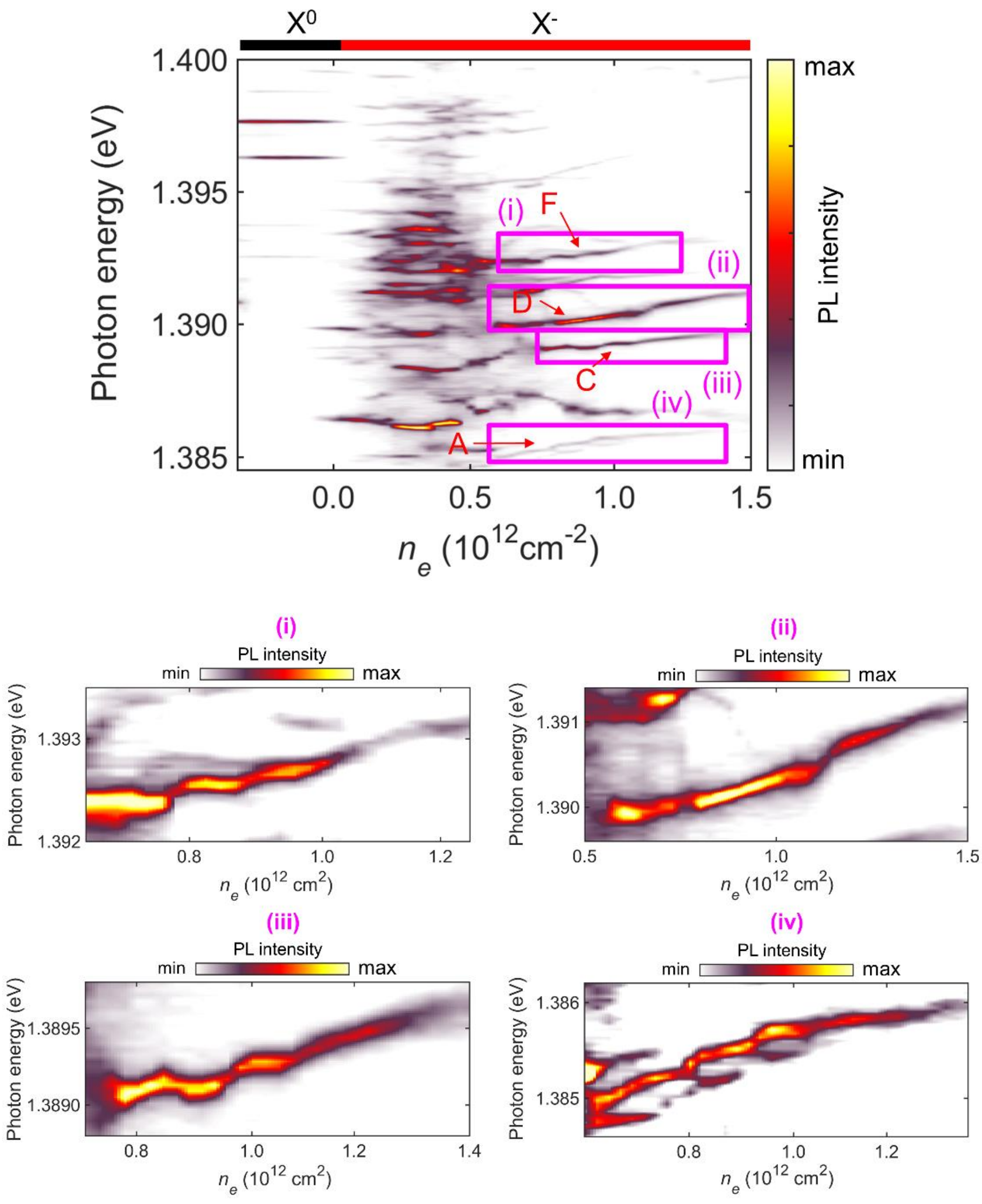

Figure S8. PL spectra of negatively charged localised IXs as a function of electron density measured at position P_1. Zoomed images for regions indicated by rectangular boxes are displayed.

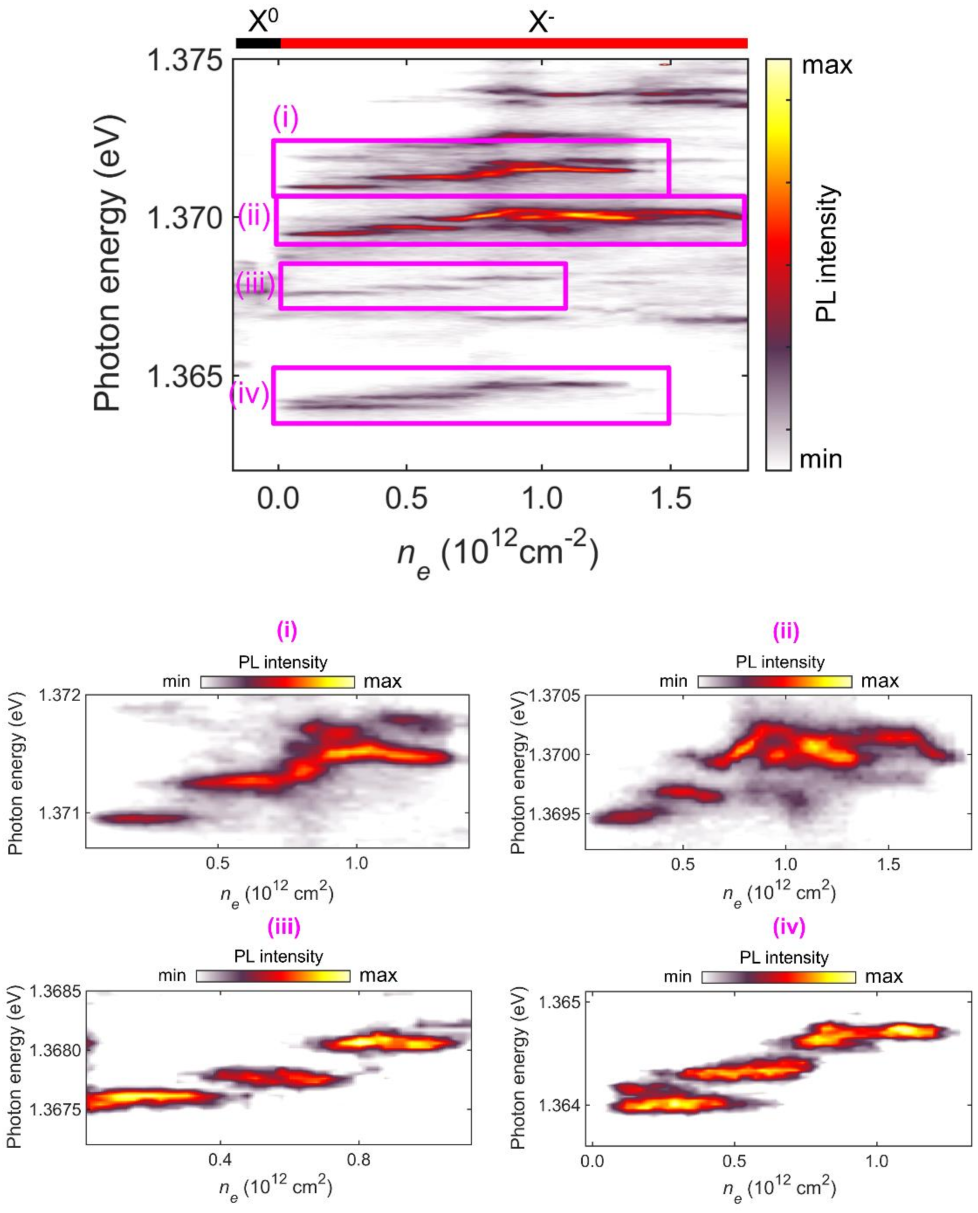

Figure S9. PL spectra of negatively charged localised IXs as a function of electron density measured at position P_2. Zoomed images for regions indicated by rectangular boxes are displayed.

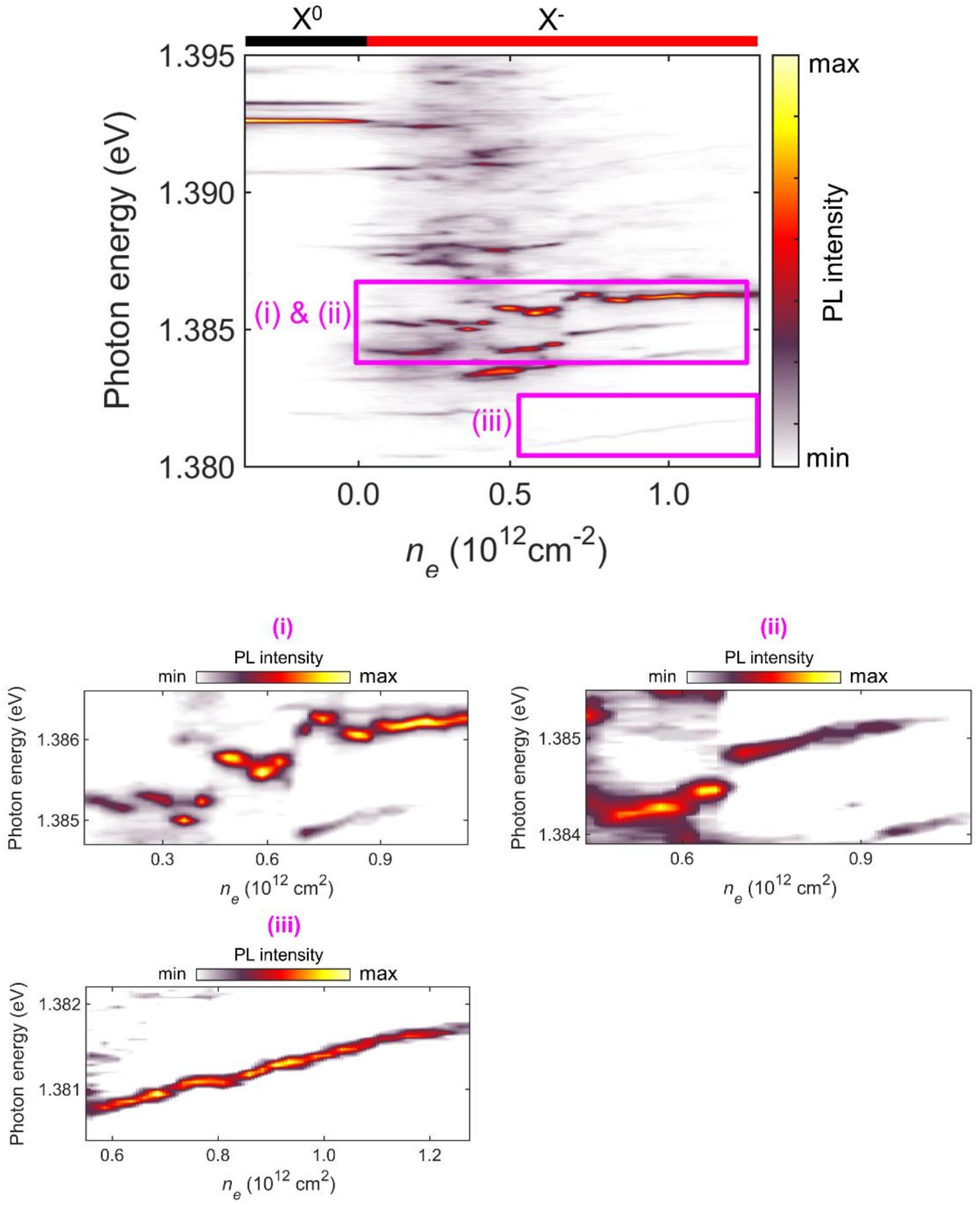

Figure S10. PL spectra of negatively charged localised IXs as a function of electron density measured at position P_3. Zoomed images for regions indicated by rectangular boxes are displayed.

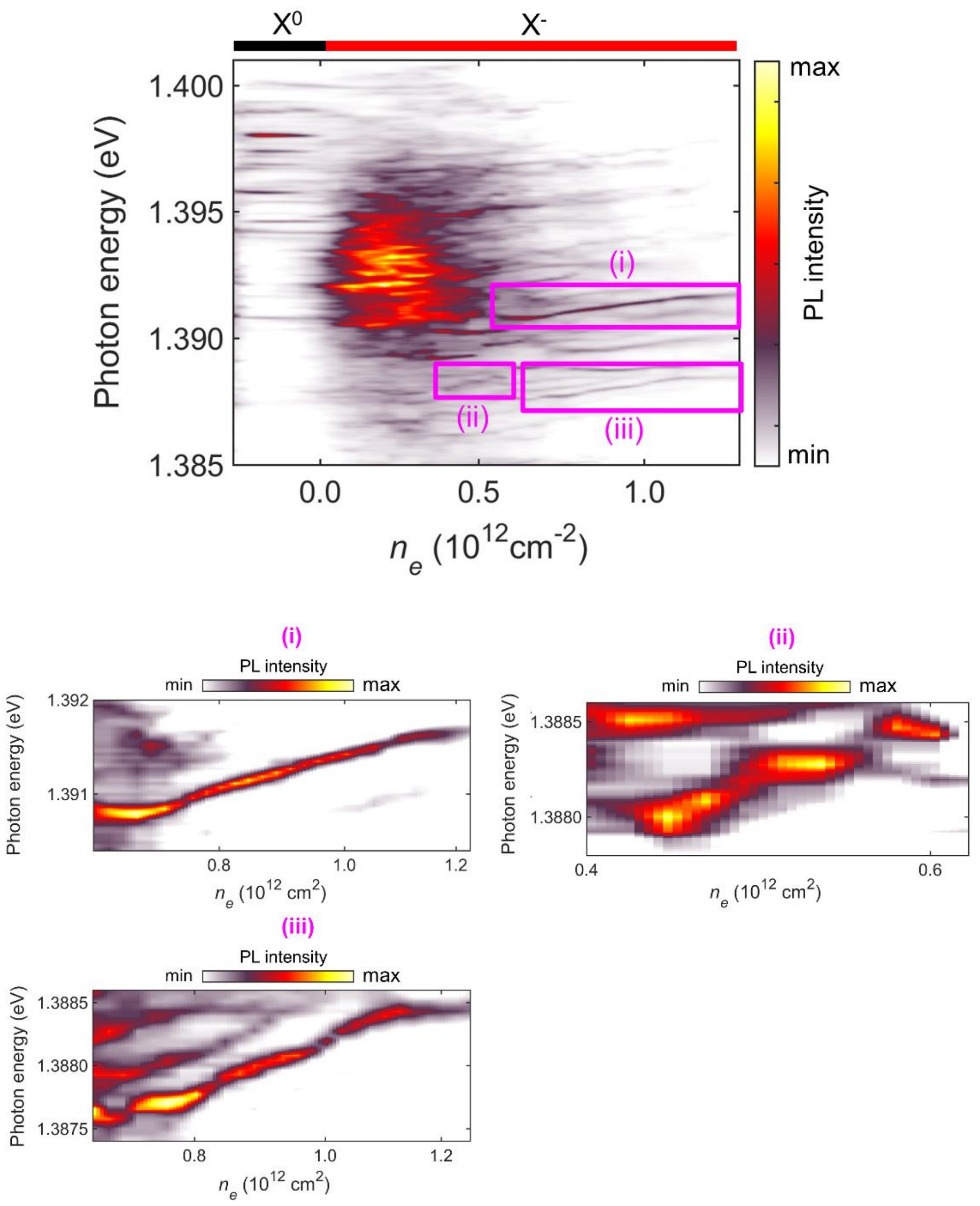

Figure S11. PL spectra of negatively charged localised IXs as a function of electron density measured at position P_4. Zoomed images for regions indicated by rectangular boxes are displayed.

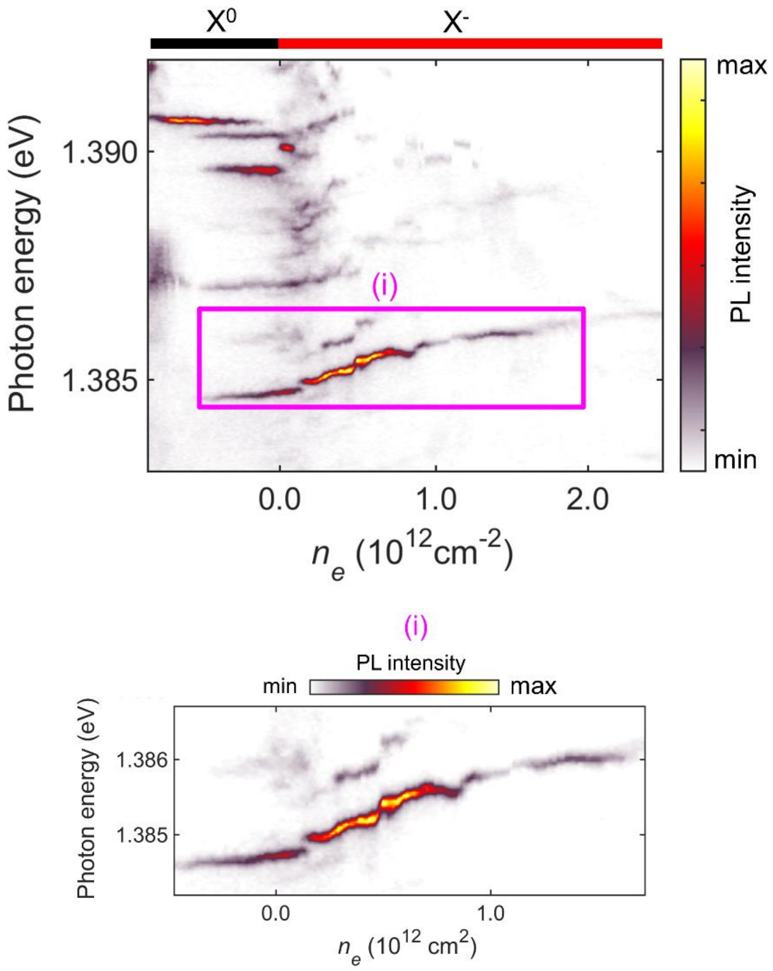

Figure S12. PL spectra of negatively charged localised IXs as a function of electron density measured at position P_5. Zoomed images for regions indicated by rectangular boxes are displayed.

Section S7. Monte Carlo simulation for discrete spectral jumps (short-range charge ordering)

As presented in Fig. 3b in the main text, U decreases rapidly as the lattice spacing increases ($\sim 1/s^2$), and thus the interaction with electrons in the nearest neighbour (NN) sites dominates over sites farther away. Therefore, we first consider only interactions with electrons in NN sites for simplicity, and the effects of outer sites will be discussed later. Figure S13(a) shows a simulated PL spectrum of a moiré-trapped IX as a function of p with w_i set to 1 for all 6 NN sites and s = 6.4 nm. As p increases, a series of spectral jumps are observed, similar to the experimentally measured data. Furthermore, the effects of varying parameters w_i and U_i (or s_i) of the NN sites are examined. Figure S13(b) shows the result when U_i of one of the sites is increased by 1.5 times compared to the other sites which might be caused by inhomogeneities in the moiré period and/or trap potential. Additional spectral peaks appear between the originally equidistant steps, leading to broader spectra for intermediate p values. Since the energy shifts are determined by the combinations of U_i of resulting from the sites filled with electrons, increasing U_i by 1.5x for one particular site leads to new possible interaction energy combinations, resulting in additional peaks. Figure S13(c) shows the simulation result when w_i for one NN site is increased from 1 to 2. The p range (plateau length) for peaks in the initial steps is reduced compared to the homogeneous case. The site with $w_i = 2$ has a higher probability of occupation, and thus it can be occupied at lower p, resulting in a shorter plateau length.

The simulated results in Figs. S13(a-c), however, show some discrepancies with the experimental results. First, in these simulation results, the intensities of the peaks at intermediate p values are weaker than the initial and final peaks. Due to the large overlap with adjacent peaks in the intermediate p range, PL intensities are distributed among the different peaks, lowering the maximum intensity. Secondly, it is hard to simulate variations between spectral jumps because a variation in U_i just results in energy broadening rather than giving different energy gaps between peaks. These discrepancies arise because the mutual interaction

between electrons in NN sites is neglected. Since the occupation probability of each site is determined independently, the overlap between the different plateaux can be enlarged without affecting the other sites in the simulation. In reality (experiment), however, the occupation probability of one site is affected by the distribution of other electrons at other sites in the moiré lattice due to the Coulomb interactions.

To address this inconsistency with experiment, another simulation is performed with different conditions. Now, it is assumed that there are preferences between neighbouring sites in the charge filling process. As recently reported, Coulomb interactions between electrons can lead to a different probability of occupation from site to site, giving rise to charge-ordered insulating states in moiré superlattices.[12,16] We also observe charge-ordered states in our sample, justifying this approach (see section S2). Furthermore, real trapping sites are not uniform, as reflected in the inhomogeneous PL spectra of moiré IXs. We therefore perform a simulation with the assumption that there is a preferential filling order between the different moiré trapping sites. To apply this assumption, the random number range used to determine the site occupancy is modified. Different ranges of random numbers, r_i $(i = 1, 2, \dots, 6)$, are set for each NN trapping sites as $0 \leq r_1 \leq 0.08$, $0.17 \leq r_2 \leq 0.25$, $0.34 \leq r_3 \leq 0.42$, $0.51 \leq r_4 \leq 0.59$, $0.68 \leq r_5 \leq 0.76$, and $0.85 \leq r_6 \leq 1$, so that trapping sites are filled with electrons in an ordered fashion.

Figure S13(d) shows the results with the ranges of r_i described above. Compared to Fig. S13(a), the overlaps between peaks are reduced, and hence, the PL intensity of each of peak becomes similar, as expected. With these simulation conditions, each spectral jump is correlated with the occupation of a specific site. In other words, the first discrete jump originates from the charge occupation at one specific site, the second one is related to the occupation at a second specific site, and so on. On the other hand, in simulations with identical r_i (Figs. S13(a)), each spectral jump arises from an equal contribution from all sites. This difference is more evident in simulations where w_i or U_i of one of the NN sites is changed. Figure S13(e) displays results when one site (site 1) has 1.5 times higher U_i. As indicated by

an arrow, only the first step is changed in contrast to the overall profile change in Fig. S13(c). Figure S13(f) shows the result when one site (site 1) has a doubled weighting factor (i.e. $w_1 = 2$ while $w_i = 1$ for $i = 2\text{–}6$). Likewise, only the first plateau step (which is related to site 1) is elongated.

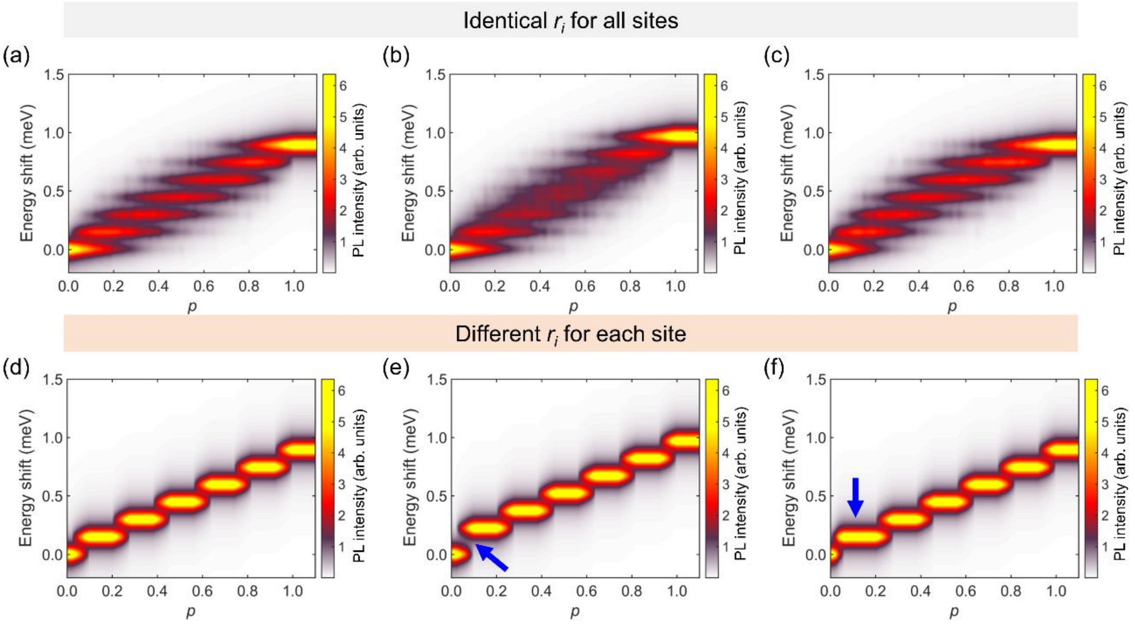

Figure S13. (a) Simulated PL maps as a function of p using the same range of random number, $0 \leq r \leq 1$ for each site. The NN sites are assumed to be homogeneous, e.g. identical w_i and U_i. **(b)** Same as (a), but U_i for one of sites is set to be 1.5 times higher than other sites. **(c)** Same as (a), but w_i for one site is set to be 2 times higher than the other sites. **(d)** Simulated PL maps as a function of p with an ordered filling process, achieved using distinctly different ranges of random numbers for each site. The NN sites are assumed to be homogenous in terms of U_i and w_i. **(e)** Same as (d), but U_i for one of sites is set to be 1.5 times higher than for other sites. **(f)** Same as (d), but w_i for one of the sites is set to be 2 times higher than for other sites. The blue arrows in (e) and (f) indicate changes in energy shift by the effect of varied U_i and w_i for one site.

In addition to NN site interaction (U_{NN}), the long-range Coulomb interactions induced by more distant sites, which we call outer sites U_{LR} ($= \sum_{i=all\ sites} U_i - \sum_{i=NN\ sites} U_i$), are considered. Figure S14(a) shows a plot of U_{LR} as a function of lattice size N for a N by N hexagonal lattice. It shows a decreasing slope as N increases. Since U is proportional to $\sim 1/s^2$, U_{LR} can be approximated to be proportional to $\sim ln\, s$, resulting in a decreasing slope with respect to s. For $N = 41$, the value of U_{LR} represents ~80% of U_{NN}. Even with a huge number of interacting sites, the increase of U_{LR} is smaller than U_{NN}. This suggests that details of the occupation process in outer sites does not alter the PL spectra substantially. Therefore, we make the assumption that the non-nearest neighbour sites are occupied without any preference (similar to the simulation shown in Fig. S13(a)), just to make the problem simpler. This assumption is added to the simulation shown in Fig. S13(d), in which NN sites were filled with an order. The result of this combination is shown in Fig. 14(b). Compared to the simulations with only NN interactions, each step shows a continuous and linear shift as p increases.

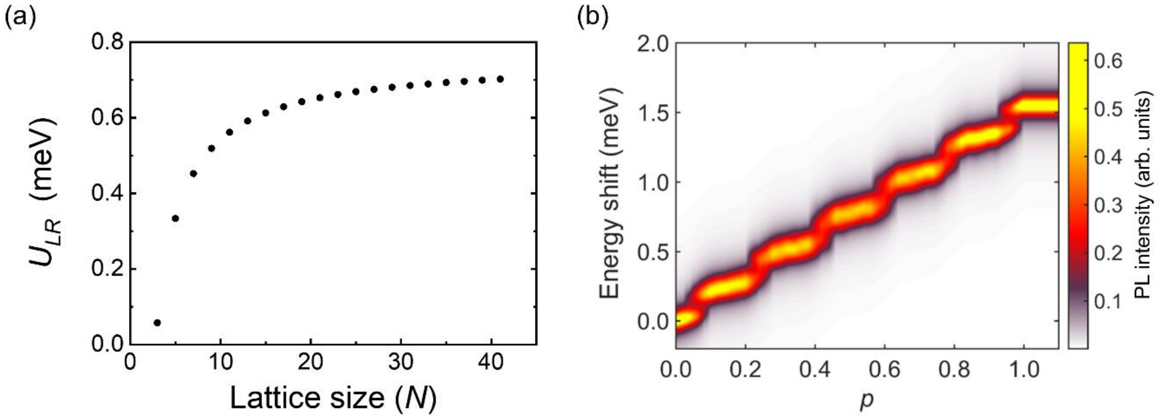

Figure S14. (a) Plot of U_{LR} versus lattice size N. **(b)** Simulated PL map with consideration of both NN and outer site interactions in a 21 by 21 hexagonal lattice. The NN sites are assumed to be occupied with an order and the non-nearest neighbour sites are set to be filled homogenously without mutual interaction.

With these simulation results, the experimentally measured PL maps can be interpreted more profoundly. First, it can be said that the discrete spectral jumps originate mainly from the filling of NN sites with a given preferential order. In addition, variations between these sites can lead to variations between spectral jumps. Second, the effect of the long-range interactions is a gradual linear blue-shift of the PL spectrum as the electron density is increased. Most commonly, the PL peaks show mostly smooth changes versus V_g, as presented in Fig. S8, S10-S12, which might result from a combination of NN and outer sites interactions. PL spectra showing unambiguous energy jumps and flat steps like the ones in Fig. 3c of the main text and in Fig. S9 are less regularly observed, and only seem to appear at special spatial positions where the NN sites trapping is exceptionally stronger than in the outer sites. Finally, in many measured PL spectra, the number of steps is less than 6, which might be related to inhomogeneity between NN sites. Some sites can have much higher or lower probability of occupation than others, so they can be pre-filled before the steps start or not filled until other sites are filled.

Section S8. Monte Carlo simulation of long-range charge-ordered states

To consider charge-ordered states, we employ a simulated annealing algorithm for classical charges on a triangular lattice.[12] The classical Monte Carlo algorithm can be thought as performing a Hartree theory calculation for the $t - U - V$ Hubbard model in the limit $t = 0, U \rightarrow \infty$. In this limit the Hamiltonian reduces to

$$H = \frac{1}{2}\sum_{\sigma,\sigma';i\neq j} V_{|i-j|} n_{i\sigma} n_{j\sigma'},$$

where $n_{i\sigma}$ is the density operator for holes on site i with spin σ. We use a Yukawa-like potential $V_{|j|} = \frac{e^2}{|j| a_m \varepsilon} e^{-|j| a_m/\rho}$ for the interaction, where ε is the background dielectric constant and ρ is the characteristic length scale of the interaction. In the classical limit or in the $T = 0$ limit, $n_{i\sigma} = \{0, 1\}$. In all our simulations we use $\varepsilon = 5$ and $\rho = \frac{2}{L}$, where L is the length of the supercell we consider. In particular, for each value of the filling factor, we construct a supercell such as νL is an integer number. We carry out simulations for several filling factors, with L ranging from 7 to 10. We also checked that using bigger supercells, e.g. $L = 15$, does not affect the lowest energy ordered state. We start our algorithm at high temperatures, such that $\frac{\Delta E^{(1)}}{k_B T_{initial}} = 0.1$, where $\Delta E^{(1)}$ is the energy difference between the initial state ($E^{(0)}$) and the first accepted state ($E^{(1)}$) (with the acceptance rule $E^1 < E^0$) and $T_{initial}$ is the initial temperature. The temperature is then decreased linearly $T^{(i+1)} = \alpha T^{(i)}$, with $\alpha = 0.95$. Between each iteration we perform $N_{MC} = 10^3 \times n_{charges}$ Monte Carlo steps at constant temperature, where $n_{charges}$ is the number of charges in the simulation. Figure S15 shows the charge-ordered states for a variety of filling factors ν (number of holes per moiré cell) that result from this procedure. We note that our results agree with those of Xu *et al.*[12].

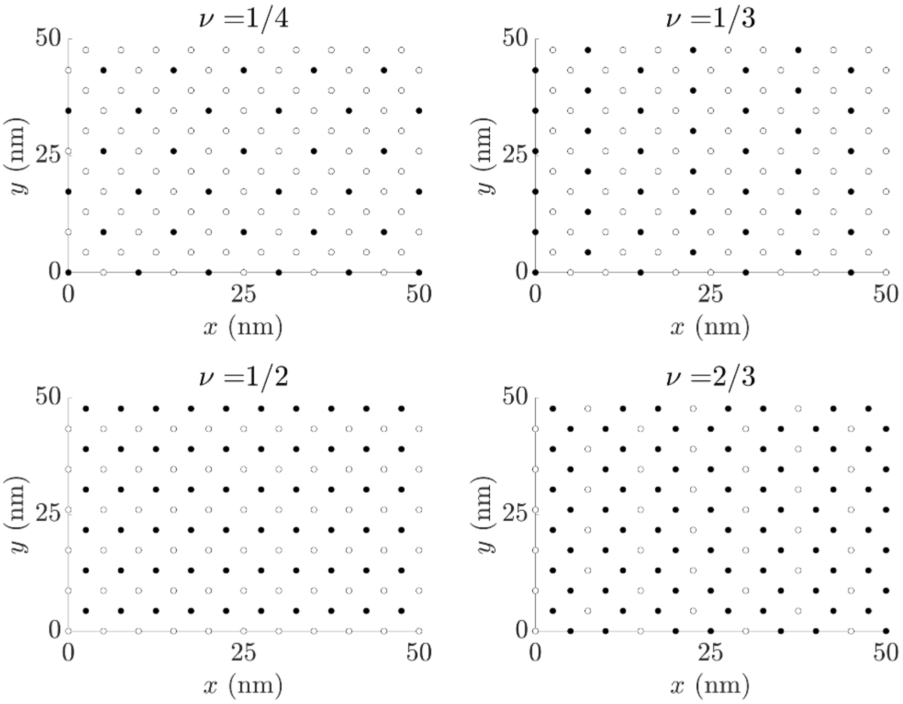

Figure S15. Charge-ordered states from classical Monte Carlo calculations for four filling factors. **(a)** $\nu = 1/4$, **(b)** $\nu = 1/3$, **(c)** $\nu = 1/2$, **(d)** $\nu = 2/3$.

For these long-range ordered charges in a moiré lattice, we consider the energy shift of the trapped IX trion. For simplicity, a random number is not introduced this time, and p is treated as the filling factor ν. Additionally, the occupation configuration is assumed to change from one ordered state to the other abruptly as p changes. Moreover, for a given long-range charge-ordered state, a trapped IX can experience up to three different immediate charge environments depending on the moiré site occupied by the IX. Interaction energies are calculated at each representative site (*S1*, *S2*, and *S3*) as indicated in Fig. S16(a) for the case of ν = 2/3. In this specific example, one can see that *S1* has 6 NN filled, while *S2* and *S3* only have 3 NN sites occupied. Figure S16(b) shows a plot of the number of filled first NN sites versus ν for each site. Figs. S16(c-e) show the calculated energy shift of the trapped IX at each site as a function of ν. Although the overall trend of a blue-shift is similar at all 3 sites, the detailed behaviour of the energy shifts varies at each site. Additionally, it is noticeable that jumps to lower energy can be observed with the long-range charge ordered states. In contrast to our prior simulations (Section S7) which included no-order or only short-range order, the mutual interaction between charges in all moiré sites considered here can result in a red-shift of the trapped IX sensor at several fractional fillings. To further refine the simulation, we also take into account the interaction of the trapped IX with charges up to the second NN sites (see Fig. S17). Although the overall trends are similar to the cases considering only the first NN sites, the inclusion of interactions with the second NN sites adds additional complexity to the energy shifts, with additional steps and energy scales.

Our results reveal red- and blue-shifted moiré-site-dependent energy shifts for different charge-ordered states when interactions with only NN and both NN and second nearest neighbours are taken into account. Additional complexity arises from the existence of three inequivalent sites in a perfect moiré lattice which yield different immediate charge environments at the same filling factor. While we see signatures of such features in the experimental data (e.g. Fig. S10), unambiguous identification of the charge-correlated states is beyond our current capabilities. In the future, this could be achieved with correlated optical reflectivity data, structural information, and perhaps the aid of machine learning techniques to identify the specific charge-correlated signatures.

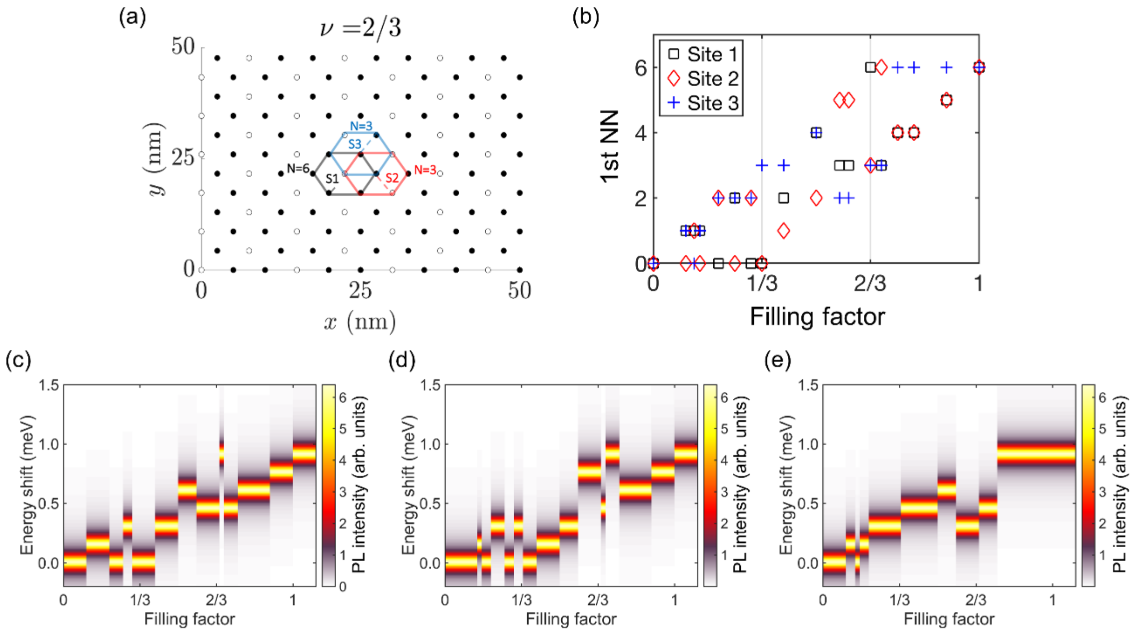

Figure S16. (a) Charge-ordered states in moiré superlattice with filling factor of 2/3. Three representative sites (*S1*, *S2*, and *S3*) and their first NN sites, in which interaction of IX and charges are considered, are indicated as black, red, and blue lines. **(b)** Plot of number of filled first NN sites versus filling factor. **(c-e)** Calculated energy shift of IX versus filling factor at *S1*, *S2*, and *S3*.

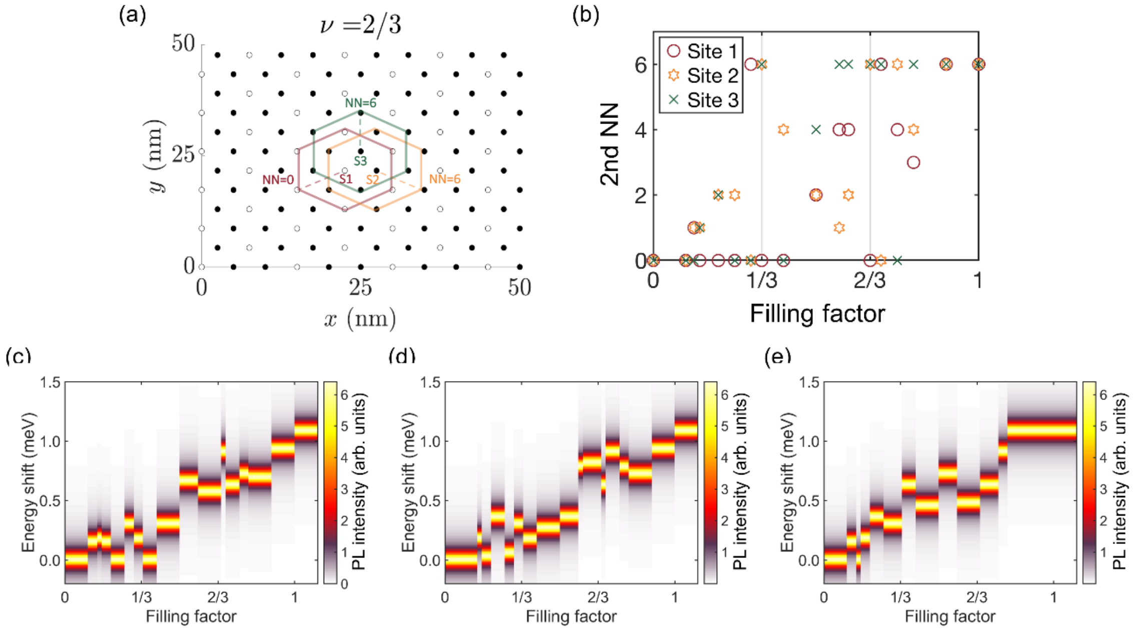

Figure S17. (a) Charge-ordered states in moiré superlattice with filling factor of 2/3. Three representative sites (*S1*, *S2*, and *S3*) and their second NN sites, in which the interaction of IX and charges are considered, are indicated as red, orange, and green lines. **(b)** Plot of the number of filled second NN sites versus filling factor. **(c-e)** Calculated energy shift of IX versus filling factor at *S1*, *S2*, and *S3*.

Section S9. Excitation energy dependence of moiré IX emission

Figure S18 shows PL maps as a function of V_g with resonant excitation for either the intralayer A-exciton of WSe_2 (1.705 eV) or the A-exciton of $MoSe_2$ (1.632 eV) at position P_1. The same data measured at position P_3 is shown in Fig. S19. First, we note some important observations: every spectral feature is reproducible, no hysteresis in the doping sweeps is present, and there are no effects of random charge noise. Further, the V_g dependence of the PL spectra is similar overall between the two different excitation energies (i.e. the trapped IX emission energies and spectral jumps are consistent), but the V_g values for each transition region from IX^0 to IX^+/IX^- are different. For $MoSe_2$ excitation, the V_g range for neutral region is shifted negative and the electron doped region is expanded compared to the WSe_2 excitation. This result indicates that excess electrons are formed via a photodoping effect when the $MoSe_2$ intralayer is resonantly excited, shifting the charge neutral region to negative V_g. Additionally, the expanded electron doped region in the PL maps for $MoSe_2$ excitation suggests that the photodoping effect is dependent on V_g. If the photodoping effect is constant, PL maps measured under different excitation energy would just show an offset of $V_{g,}$ which is in contrast to the observed data. The experimental results indicate the photodoping effect decreases a V_g increases in the positive direction.

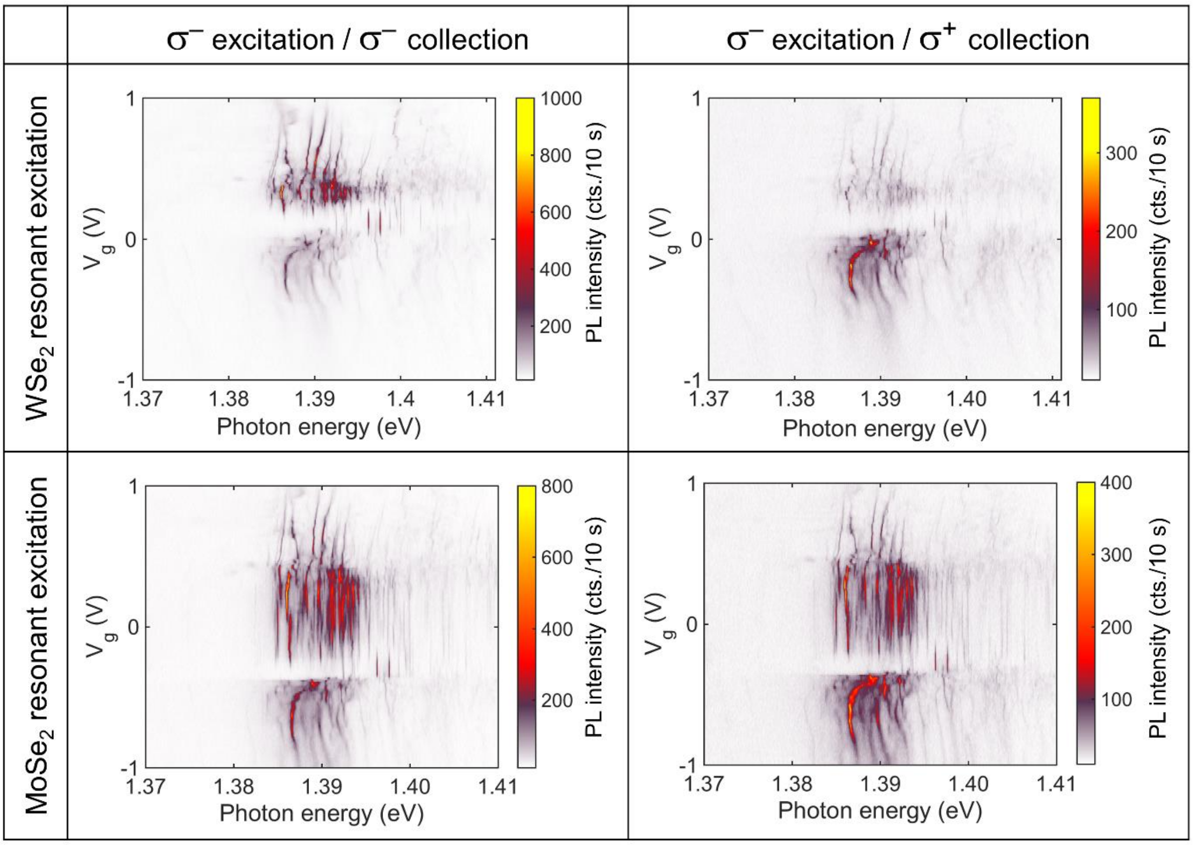

Figure S18. Co- and cross-polarised PL maps as a function of V_g with resonant excitation to the intralayer A-exciton of WSe_2 (1.705 eV) and $MoSe_2$ (1.632 eV) measured at position P_1.

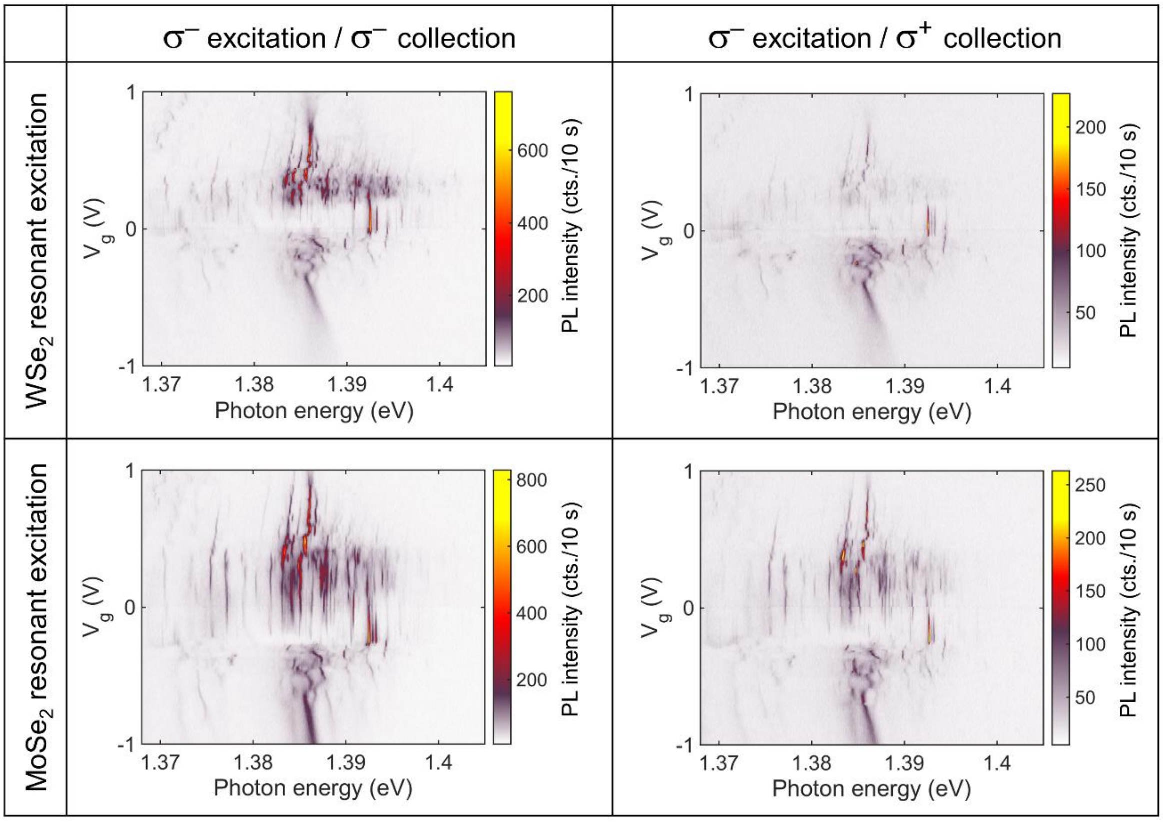

Figure S19. Co- and cross-polarised PL maps as a function of V_g with resonant excitation to intralayer A-excitons of WSe_2 (1.705 eV) and $MoSe_2$ (1.632 eV) measured at position P_3.

Section S10. DOCP map with resonant excitation to the intralayer A-exciton of $MoSe_2$ (1.632 eV)

Figures S20(a) and (c) show DOCP maps as a function of V_g measured with resonant excitation to the intralayer A-exciton of WSe_2 and $MoSe_2$, respectively. The data in Fig. S20(a) is the same one shown in Fig. 4a in the main text. The excitation laser is σ^- polarised. Representative PL spectra collected with $\sigma^\pm$ polarisation at different doping conditions are presented in Figs. S20(b) and (d). The overall dependence is similar for the two different excitation energies, though the exact DOCP values are not the same. In both excitation cases, IX^0 and IX^- show strongly co-polarised emission and the DOCP abruptly reduces to nearly 0 with hole doping as explained in main text. The abrupt decrease of DOCP in the hole doped region even under resonant excitation to $MoSe_2$ indicates that the excitation valley polarisation is predominantly maintained through a hole in WSe_2. In addition, the DOCP for IX^0 and IX^- are lower under $MoSe_2$ resonant excitation compared to WSe_2 resonant excitation. With the $MoSe_2$ excitation, holes generated in $MoSe_2$ layer transfer rapidly to the WSe_2 layer due to the band alignment. Since valley polarisation is predominantly maintained through a hole in WSe_2, the lower value of DOCP for $MoSe_2$ excitation reveals that valley scattering processes occur during this charge transfer process.

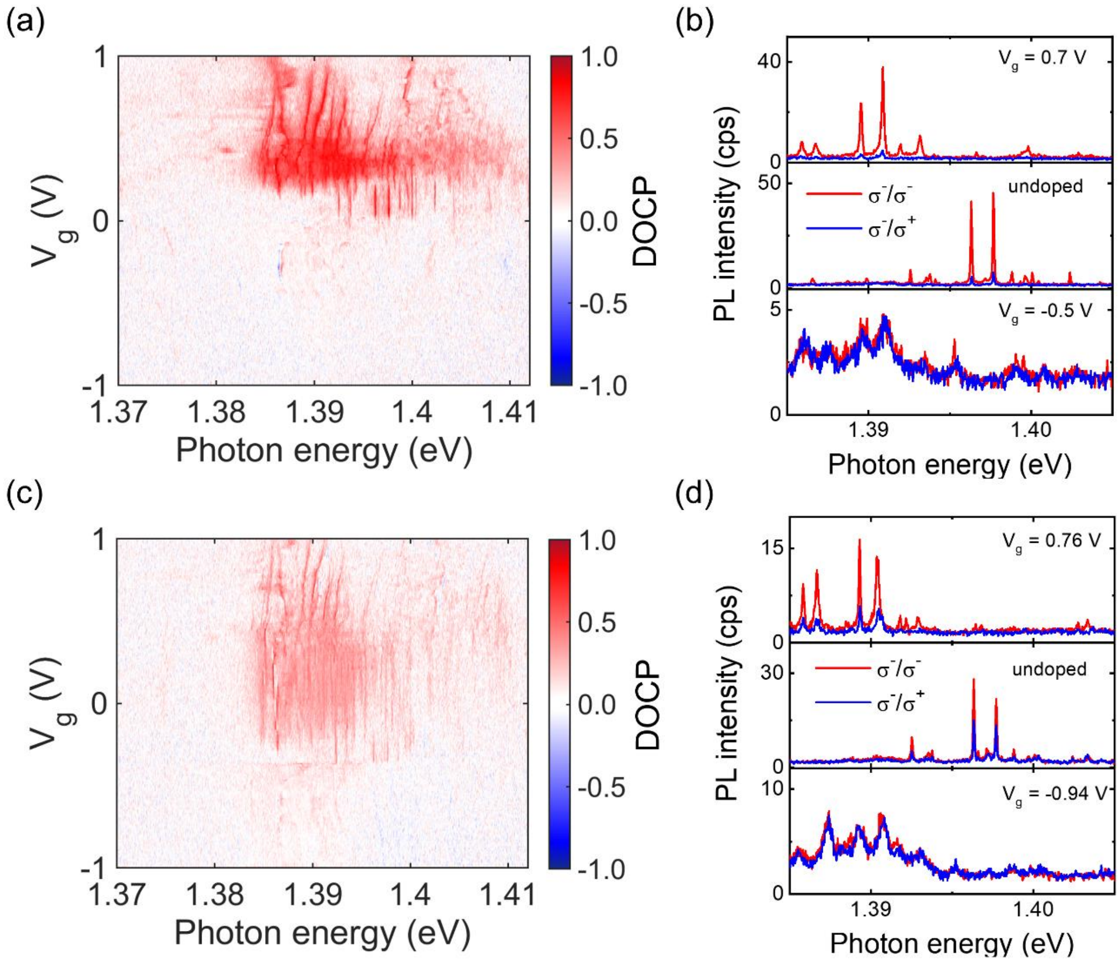

Figure S20. (a,c) DOCP maps as a function of V_g with resonant excitation for WSe_2 and $MoSe_2$ respectively. **(b,d)** Polarisation-resolved PL spectra at different doping conditions. The spectra for the red and blue lines represent co- and cross-polarised excitation/emission, respectively.

Section S11. Energy band diagrams for IX for an applied B-field

Figure S21 shows electron energy band diagrams for the H-stacked $MoSe_2/WSe_2$ heterobilayer for an applied B-field. The shift of the conduction and valence band edge energy (ΔE_c and ΔE_v) with B-field can be expressed as

$$\Delta E_c = (2S_z + \tau\alpha_c)\mu_B B$$

$$\Delta E_v = (2S_z + m_l + \tau\alpha_v)\mu_B B$$

where μ_B is the Bohr magneton, $S_z = \pm 1/2$ is the electron spin value, $\tau = \pm 1$ is the valley index, $m_l = \pm 2$ is the magnetic quantum number for the atomic orbital at the valence band edge and $\alpha_{c,v}$ is the valley magnetic moment for the conduction/valence band.[17] According to a $k \cdot p$ theory, $\alpha_{c,v} = m_0/m^*_{c,v}$ where m_0 is the free electron mass and $m^*_{c,v}$ is the electron/hole effective mass at the conduction/valence band edges.[18] As a result, the magnitude of the energy difference in the conduction and valence bands between valleys (Δ_c and Δ_v) are

$$\Delta_c = (2 + 2\alpha_c)\mu_B B$$

$$\Delta_v = (2 + 4 + 2\alpha_v)\mu_B B.$$

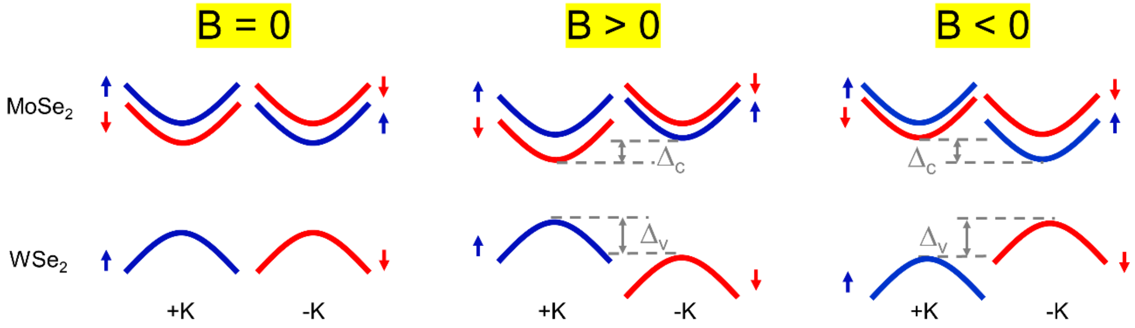

Figure S21. Electron energy band diagrams for the H-type stacked $MoSe_2/WSe_2$ heterobilayer for different B-field conditions.

References

1 Back, P. *et al.* Giant paramagnetism-induced valley polarization of electrons in charge-tunable monolayer $MoSe_2$. *Phys. Rev. Lett.* **118**, 237404 (2017).

2 Wang, Z., Zhao, L., Mak, K. F. & Shan, J. Probing the spin-polarized electronic band structure in monolayer transition metal dichalcogenides by optical spectroscopy. *Nano Lett.* **17**, 740-746 (2017).

3 Efimkin, D. K. & MacDonald, A. H. Many-body theory of trion absorption features in two-dimensional semiconductors. *Phys. Rev. B* **95**, 035417 (2017).

4 Sidler, M. *et al.* Fermi polaron-polaritons in charge-tunable atomically thin semiconductors. *Nat. Phys.* **13**, 255-261 (2017).

5 Courtade, E. *et al.* Charged excitons in monolayer WSe_2: Experiment and theory. *Phys. Rev. B* **96**, 085302 (2017).

6 Hong, X. *et al.* Ultrafast charge transfer in atomically thin MoS_2/WS_2 heterostructures. *Nat. Nanotechnol.* **9**, 682-686 (2014).

7 Wilson, N. R. *et al.* Determination of band offsets, hybridization, and exciton binding in 2D semiconductor heterostructures. *Sci. Adv.* **3**, e1601832 (2017).

8 Jin, C. *et al.* Imaging of pure spin-valley diffusion current in WS_2-WSe_2 heterostructures. *Science* **360**, 893-896 (2018).

9 Brem, S., Linderälv, C., Erhart, P. & Malic, E. Tunable Phases of Moiré Excitons in van der Waals Heterostructures. *Nano Lett.* **20**, 8534-8540 (2020).

10 Wu, F., Lovorn, T. & MacDonald, A. H. Topological exciton bands in moiré heterojunctions. *Phys. Rev. Lett.* **118**, 147401 (2017).

11 Tang, Y. *et al.* Simulation of Hubbard model physics in WSe_2/WS_2 moiré superlattices. *Nature* **579**, 353-358 (2020).

12 Xu, Y. *et al.* Correlated insulating states at fractional fillings of moiré superlattices. *Nature* **587**, 214-218 (2020).

13 Brixner, L. Preparation and properties of the single crystalline AB2-type selenides and tellurides of niobium, tantalum, molybdenum and tungsten. *J. Inorg. Nucl. Chem.* **24**, 257-263 (1962).

14 Jin, C. H. *et al.* Observation of moire excitons in WSe_2/WS_2 heterostructure superlattices. *Nature* **569**, 76-80 (2019).

15 Liu, E. *et al.* Excitonic and valleytronic signatures of correlated states at fractional fillings of a moiré superlattice. *https://arxiv.org/abs/2011.13892* (2020).

16 Regan, E. C. *et al.* Mott and generalized Wigner crystal states in WSe_2/WS_2 moiré superlattices. *Nature* **579**, 359-363 (2020).

17 Aivazian, G. *et al.* Magnetic control of valley pseudospin in monolayer WSe_2. *Nat. Phys.* **11**, 148-152 (2015).

18 Yao, W., Xiao, D. & Niu, Q. Valley-dependent optoelectronics from inversion symmetry breaking. *Phys. Rev. B* **77**, 235406 (2008).
